# Postnatal Osterix but not DMP1 lineage cells significantly contribute to intramembranous ossification in three preclinical models of bone injury

**DOI:** 10.3389/fphys.2022.1083301

**Published:** 2023-01-04

**Authors:** Evan G. Buettmann, Susumu Yoneda, Pei Hu, Jennifer A. McKenzie, Matthew J. Silva

**Affiliations:** ^1^ Department of Orthopaedic Surgery, Washington University in St. Louis School of Medicine, St. Louis, MO, United States; ^2^ Department of Biomedical Engineering, Washington University in St. Louis, St. Louis, MO, United States; ^3^ Department of Biomedical Engineering, Virginia Commonwealth University, Richmond, VA, United States

**Keywords:** osteoblast lineage cells, fracture healing, lineage tracing, stress fracture, osteoprogenitor cells, inducible Cre-LoxP recombination

## Abstract

Murine models of long-bone fracture, stress fracture, and cortical defect are used to discern the cellular and molecular mediators of intramembranous and endochondral bone healing. Previous work has shown that Osterix (Osx^+^) and Dentin Matrix Protein-1 (DMP1^+^) lineage cells and their progeny contribute to injury-induced woven bone formation during femoral fracture, ulnar stress fracture, and tibial cortical defect repair. However, the contribution of pre-existing *versus* newly-derived Osx^+^ and DMP1^+^ lineage cells in these murine models of bone injury is unclear. We addressed this knowledge gap by using male and female 12-week-old, tamoxifen-inducible Osx Cre_ERT2 and DMP1 Cre_ERT2 mice harboring the Ai9 TdTomato reporter allele. To trace pre-existing Osx^+^ and DMP1^+^ lineage cells, tamoxifen (TMX: 100 mg/kg gavage) was given in a pulse manner (three doses, 4 weeks before injury), while to label pre-existing and newly-derived lineage Osx^+^ and DMP1^+^ cells, TMX was first given 2 weeks before injury and continuously (twice weekly) throughout healing. TdTomato positive (TdT^+^) cell area and cell fraction were quantified from frozen histological sections of injured and uninjured contralateral samples at times corresponding with active woven bone formation in each model. We found that in uninjured cortical bone tissue, Osx Cre_ERT2 was more efficient than DMP1 Cre_ERT2 at labeling the periosteal and endosteal surfaces, as well as intracortical osteocytes. Pulse-labeling revealed that pre-existing Osx^+^ lineage and their progeny, but not pre-existing DMP1^+^ lineage cells and their progeny, significantly contributed to woven bone formation in all three injury models. In particular, these pre-existing Osx^+^ lineage cells mainly lined new woven bone surfaces and became embedded as osteocytes. In contrast, with continuous dosing, both Osx^+^ and DMP1^+^ lineage cells and their progeny contributed to intramembranous woven bone formation, with higher TdT^+^ tissue area and cell fraction in Osx^+^ lineage *versus* DMP1^+^ lineage calluses (femoral fracture and ulnar stress fracture). Similarly, Osx^+^ and DMP1^+^ lineage cells and their progeny significantly contributed to endochondral callus regions with continuous dosing only, with higher TdT^+^ chondrocyte fraction in Osx^+^ *versus* DMP1^+^ cell lineages. In summary, pre-existing Osx^+^ but not DMP1^+^ lineage cells and their progeny make up a significant amount of woven bone cells (particularly osteocytes) across three preclinical models of bone injury. Therefore, Osx^+^ cell lineage modulation may prove to be an effective therapy to enhance bone regeneration.

## Introduction

Bone is one of the only tissues in the body that can heal with scarless tissue regeneration. This remarkable capacity for self-repair requires a complex, multi-faceted process that involves growth factors, mechanical cues, and unique populations of cells. Based on these environmental factors, bone healing occurs either *via* endochondral or intramembranous ossification. In endochondral ossification, progenitor cells first differentiate and form a cartilage callus that is later replaced by bone. In contrast, intramembranous ossification results in direct bone formation from progenitor cells, bypassing the cartilage intermediate. Although still unclear, studies indicate that endochondral processes are favored in environments with low oxygen tension, vascular disruption, and some micromotion (non-rigid fixation) (Toyosawa et al., 2004; Marsell and Einhorn, 2011; Bahney et al., 2015; Miller et al., 2015). With nearly 5–10% of fractures progressing to delayed healing or non-union (Woolf and Pfleger, 2003; Einhorn and Gerstenfeld, 2015) and resulting in increased medical cost and loss of productivity (Bonafede et al., 2013), understanding the cellular and molecular mediators of both and endochondral and intramembranous ossification following bone injury is paramount.

Preclinical models of bone injury are critical for dissecting the cellular and molecular processes controlling endochondral and intramembranous ossification. The most common injury model used is the transverse, full fracture (“Einhorn model”) first developed by Bonnarens and Einhorn (Bonnarens and Einhorn, 1984). This model has been adapted for use in both the tibia and femur of rats and mice (Bonnarens and Einhorn, 1984; An et al., 1994; Zondervan et al., 2018; Buettmann et al., 2019), and utilizes blunt trauma to induce a mid-diaphyseal fracture that is stabilized with an intramedullary rod. Due to the semi-stable nature of fixation, this model heals by periosteal intramembranous woven bone formation near the callus periphery and endochondral ossification near the fracture site, with both woven bone tissue and cartilage visible by day 14 post-injury (Colnot, 2009; Buettmann et al., 2019). Tissue transplantation studies have determined that cells from the periosteum are the primary contributors to callus formation in this model, with smaller contributions from the adjacent skeletal muscle and marrow (Colnot, 2009; Julien et al., 2022). In contrast, the rodent stress fracture model, developed and characterized in our lab, utilizes forelimb cyclic fatigue loading to create a non-displaced ulnar fracture that heals predominantly by periosteal intramembranous woven bone formation 10–14 days post-injury (Hsieh and Silva, 2002; Uthgenannt et al., 2007; Wohl et al., 2009; Martinez et al., 2010). Bulk RNAseq analysis comparing the transverse, full fracture model *versus* stress fracture model in mice indicates that the stress fracture model has a shorter, less pronounced inflammatory phase and a more enriched osteogenic signature (Coates et al., 2019). Another widely used bone repair model is the monocortical defect injury. In this model, a small monocortical defect (0.4–0.8 mm in diameter) is drilled in the mid-diaphysis of the long-bone (Liu et al., 2018; Buettmann et al., 2019; Li and Helms, 2021). Healing progresses after injury with inflammation followed by small amounts of periosteal cartilage callus formation between days 3 and 7 (Hu and Olsen, 2016; Liu et al., 2018). By days 5–10 after injury, intramedullary intramembranous hard callus formation occurs, followed by resolution at days 14–21. Due to the differing healing modalities among these three bone injury models, their simultaneous utilization can provide insights into the unique cellular and molecular mediators of bone healing (Supplementary Figure S1).

Tracking the cellular mediators of bone healing has been aided by the recent development of many tamoxifen-inducible Cre constructs (Cre_ERT2) that can be crossed with fluorescent transgenic reporters (Ai9, Ai14, mTmG, YFP, etc.), allowing for longitudinal tracking of targeted cell populations that contribute to fracture healing *in vivo* (Feil et al., 2009; Madisen et al., 2010; Abe and Fujimori, 2013; Seime et al., 2015). The emerging role of different skeletal stem cells in fracture repair has been reported by numerous groups and was reviewed recently (Serowoky et al., 2020). We have focused on cells at the later stage of the osteoblast lineage (Osx and later), and used continuous tamoxifen dosing to demonstrate that Osx^+^ lineage cells and their progeny (labeled in Osterix Cre_ERT2 (Maes et al., 2010) Ai9 (Madisen et al., 2010) mice) contributed greater cell numbers than DMP1^+^ lineage cells (labeled in Dentin Matrix-Protein 1 Cre_ERT2 (Powell et al., 2011) Ai9 (Madisen et al., 2010) mice) and their progeny to woven bone formation in femoral transverse, ulnar stress fracture, and tibial cortical defects (Buettmann et al., 2019). However, because cells were labeled before and during healing by continuous tamoxifen, we could not determine the contribution of pre-existing *versus* newly differentiated Osx^+^ and DMP1^+^ lineage cells and their progeny (herein labeled Osx^+^ or DMP1^+^ lineage cells) to fracture callus tissues. More recent work by our lab group used pulse-chase labeling strategies and demonstrated that pre-existing Osx^+^ and DMP^+^ lineage cells and their progeny contribute significantly to early lamellar bone formation following anabolic (non-damaging) skeletal loading (Zannit and Silva, 2019; Harris and Silva, 2022). Interestingly, we observed that these lineage-labeled cells, especially DMP1^+^ lineage cells, are rapidly depleted from the periosteal bone surface when a higher loading stimulus induces woven bone formation (Zannit and Silva, 2019; Harris and Silva, 2022). Together these data indicate that Osx^+^ and DMP1^+^ lineage cells play a role in load-induced bone formation and bone healing, however the relative contributions of pre-existing *versus* newly-derived Osx^+^ and DMP1^+^ lineage cells and their progeny across various bone injury types remains poorly defined.

Using both continuous and pulse-chase tamoxifen dosing strategies, we sought to determine the role of pre-existing and/or newly-differentiated Osx^+^ and DMP1^+^ lineage cells and their progeny in three pre-clinical models of bone repair: transverse femoral fracture, ulnar stress fracture and tibial cortical defect (Supplementary Figure S1). Due to the wider resident bone cell population reported to be targeted with Osx Cre_ERT2 construct (Maes et al., 2010), we hypothesized, that pre-existing Osx^+^ lineage cells target a greater portion of woven bone regions *versus* pre-existing DMP1^+^ lineage across all three injury models. Furthermore, we hypothesized that lineage-labeled cells in woven bone callus would be significantly increased with continuous dosing compared to pulse dosing in both Osx Cre_ERT2 Ai9 and DMP1 Cre_ERT2 Ai9 mice.

## Methods

### Mouse lines

**FIGURE 1:**
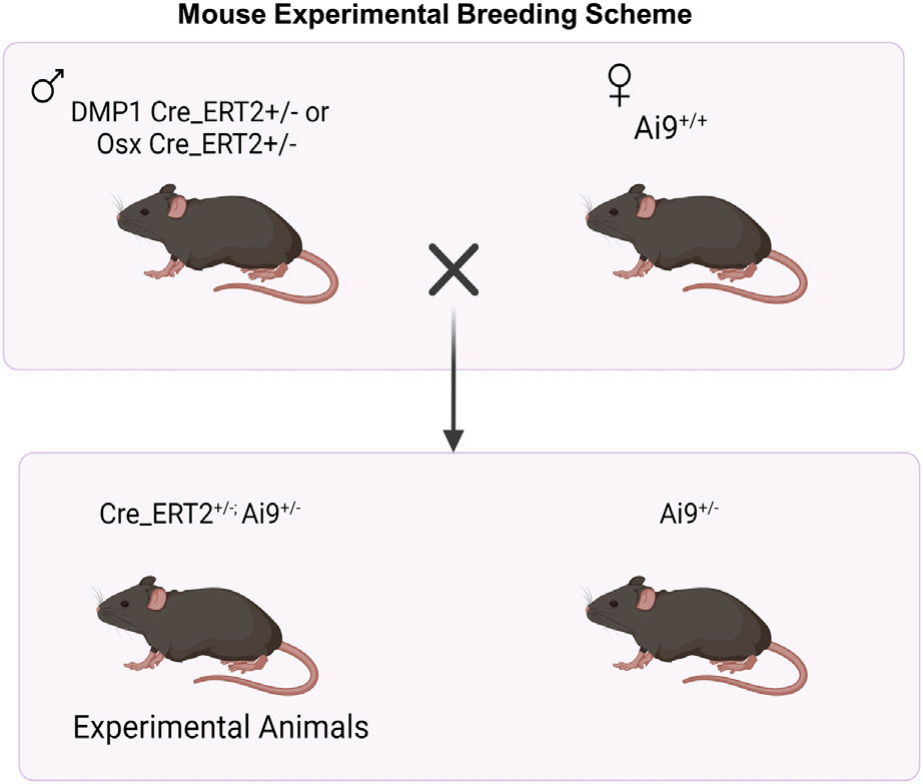
Breeding Strategy for Osx^+^ and DMP1^+^ Osteoblast Cell Lineage Analysis. For experimental animal generation, male mice hemizygous for inducible Cre (Cre_ERT2^+/−^) were crossed to female mice containing homozygous Ai9 alleles (Ai9^+/+^). Male and female mice hemizygous for Osx or DMP1 Cre_ERT2 and containing the Ai9 allele were utilized for experiments. + = presence of transgene; - = absence of transgene. Figure created in Biorender.

All mouse breeding and experimental protocols were approved by Washington University in St. Louis IACUC. Mouse lines including Osx Cre_ERT2 (Maes et al., 2007), DMP1 Cre_ERT2 (Powell et al., 2011), and Ai9 (RCL-tdTomato) (Madisen et al., 2010) were previously generated and described. Osx Cre_ERT2 and DMP1 Cre_ERT2 breeders were shared from the laboratories of Drs. Henry Kronenberg and Paola Pajevic, respectively. Ai9 (RCL-tdT; Catalog #007909) breeders were purchased from Jackson Laboratories. All mice were obtained from a previously backcrossed C57BL/6J line. To generate inducible Cre reporter mice, male mice hemizygous for Cre were crossed to female mice containing homozygous Ai9 alleles (Figure 1).

### Experimental overview and tamoxifen dosing timeline

**FIGURE 2:**
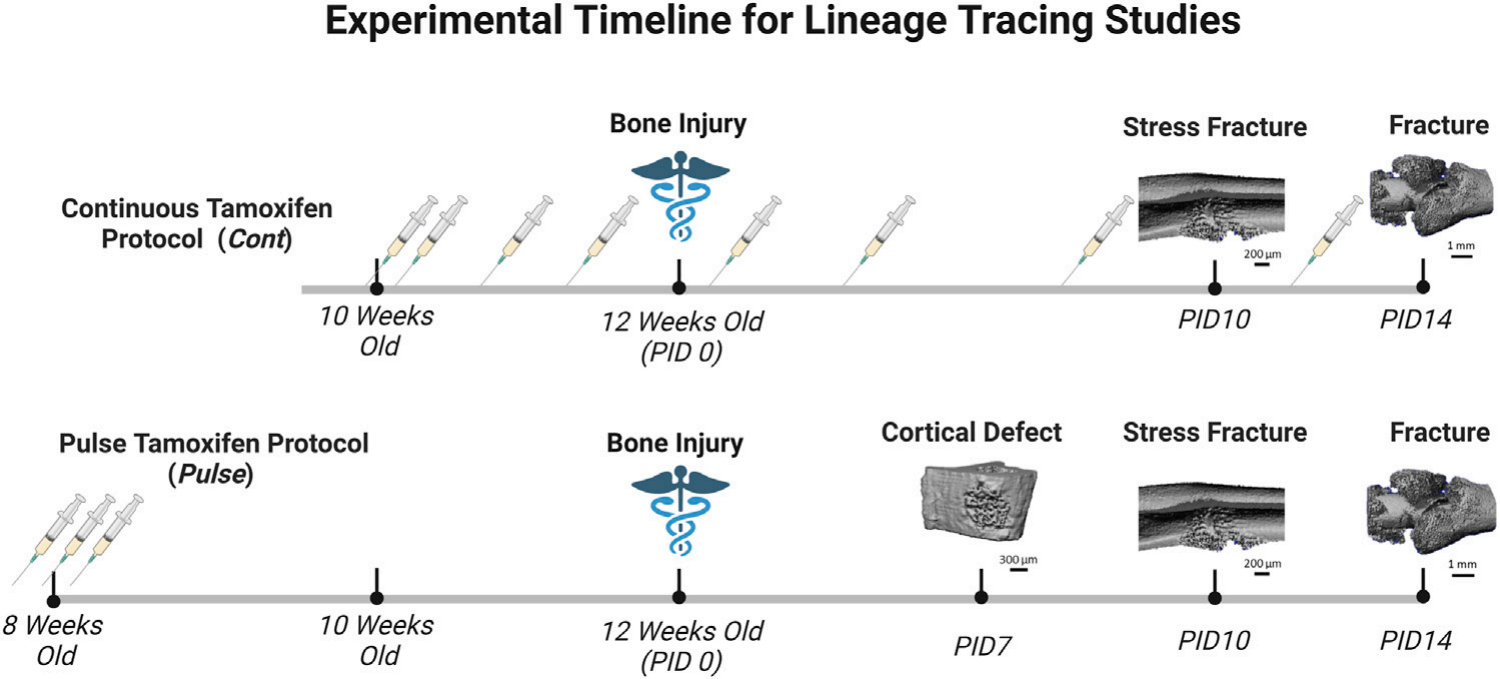
Timeline of Pulse Versus Continuous Dosing During Bone Repair. Mice were either given tamoxifen by oral gavage (syringe icon in Figure 2) (TMX; 100 mg/kg) continuously (2 days/wk) starting at 10 weeks old, or pulsed with TMX for three consecutive days starting at 8 weeks old. Then mice underwent bone injury *via* semi-stabilized femoral fracture, ulnar stress fracture or tibial defect injury at 12 weeks. Mice were sacrifice at pre-defined timepoints corresponding with robust woven bone formation for each injury model (microCT reconstruction for woven bone; day 7 defect; day 10 stress fracture; day 14 femoral fracture). Days post-injury (PID) are subsequently labeled. Figure created in Biorender.

Tamoxifen (TMX) was mainly administered by oral gavage dissolved in corn oil (Sigma‐Aldrich, CAS #10540-29‐1; 100 mg/kg). In an initial cohort of mice (∼10% of study), TMX was given by chow diet (ENVIGO TD. 130859; ∼40 mg/kg daily) for continuous dosing strategies but later discontinued in favor of gavage dosing. We did not observe differences in TdTomato expression during bone healing between tamoxifen administration methods when used continuously (data not shown). Experimental mice harboring Osx Cre_ERT2^+/−^; Ai9^+/−^ (^+^ = presence of transgene;^−^= absence of transgene) or DMP1 Cre_ERT2^+/−^; Ai9^+/−^ and given TMX served as Cre reporter mice and are labeled as Osx^TMX^ and DMP1^TMX^, respectively (Table 1). Mice harboring Cre and Ai9 alleles and only given the vehicle corn oil or chow without tamoxifen were used to assess Cre non-inducible recombination (i.e. “leakiness”) and are labeled as Osx^VEH^ or DMP1^VEH^, respectively. To label pre-existing Osx^+^ and DMP1^+^ lineage cells (and their progeny) as well as newly-derived Osx^+^ and DMP1^+^ lineage cells and their progeny after bone injury, mice were given TMX continuously (2x weekly; 100 mg/kg) starting at 2 weeks before injury and throughout healing (Figure 2). These mice are referred to as Osx^TMX^;Continuous and DMP1^TMX^;Continuous groups, respectively. To label only pre-existing Osx^+^ and DMP1^+^ lineage cells (and their progeny), mice were given three TMX doses 4 weeks before bone injury at 8 weeks of age (Figure 2). These mice are referred to as Osx^TMX^;Pulse and DMP1^TMX^;Pulse groups, respectively. We have previously reported residual tamoxifen effects on bone formation are negligible following a 4-week clearance time (Zannit and Silva, 2019). Male and female mice were used as available and in approximately equal numbers among experimental groups. We utilized both males and females in this study since both mouse sexes have been readily utilized in these inducible Cre lines in previous literature (Buettmann et al., 2019; McKenzie et al., 2019; Harris and Silva, 2022). Mice were group housed under a standard 12-h light/dark cycle and given access to food and water *ad libitum*.

**TABLE 1:** Overview of experimental groups.

Mouse genotype	Treatment(100 mg/kg)	Abbreviation
Osx Cre_ERT2^+/−^; Ai9^+/−^	Tamoxifen in Corn Oil	Osx^TMX^
Osx Cre_ERT2^+/−^; Ai9^+/−^	Vehicle (Corn Oil)	Osx^VEH^
DMP1 Cre_ERT2^+/−^; Ai9^+/−^	Tamoxifen in Corn Oil	DMP1^TMX^
DMP1 Cre_ERT2^+/−^; Ai9^+/−^	Vehicle (Corn Oil)	DMP1^VEH^

### Models of bone repair

For each bone repair model, the right limb was injured whereas the left contralateral limb served as the uninjured control. Mice were given buprenorphine SR LAB (1 mg/kg, s. c.) one hour before injury, and anesthetized during all procedures with isoflurane (1–3% v/v). The right limb was shaved and sterilized with betadine and alcohol (70%) before surgery. Following all bone injury procedures, mice were returned to their cage and placed on electronic heating pads (BeanFarm; Ultratherm) until awake and sternal. Mice were monitored daily for signs of pain and distress and open wounds were quickly resutured and treated with topical triple antibiotic ointment.

#### Femoral semi-stabilized transverse fracture

Right femurs were prepared for fracture as previously described (McBride-Gagyi et al., 2015; McKenzie et al., 2018). Briefly, a complete (full) transverse bone fracture was made in the femoral mid-diaphysis *via* three-point bending using a custom designed fixture on a materials testing machine (Instron, DynaMight 8841). The fracture was stabilized with a 24-gauge stainless steel pin (Microgroup, #304 H24RW) and the wound sutured with 3–0 nylon sutures in a simple interrupted pattern (Ethicon, #1669H). Immediately after fracture, lateral radiographs at ×3 magnification (Faxitron, Ultrafocus 100) were taken to verify proper fixation of the fracture site. Mice were allowed to heal for 14 days post-injury (PID 14), when the intramembranous woven bone on the callus periphery and cartilage undergoing endochondral ossification near the fracture line are both visible (Supplementary Figure S1) (Einhorn and Gerstenfeld, 2015; Buettmann et al., 2019).

#### Ulnar stress fracture

Right ulnas had a stress fracture generated as previously described (Martinez et al., 2010; Buettmann et al., 2019). Briefly, a non-displaced (partial) stress fracture was made in the ulnar mid-diaphysis *via* fatigue loading by cyclic compression on a material testing machine (Instron, DynaMight 8841). Right forelimbs were loaded at a calibrated peak force of 3.1 N to a 50% increase in cyclic displacement from the 10th cycle of loading. Previous work has shown that loading to this average cyclic displacement level in similarly aged wildtype C57BL/6J mice produces a reproducible non-displaced crack on the compressive surface (Buettmann et al., 2019). Mice were allowed to heal for 10 days post-injury (PID 10), when the woven bone response, predominantly formed *via* periosteal intramembranous ossification, is maximal (Supplementary Figure S1) (Uthgenannt et al., 2007; Martinez et al., 2010).

#### Tibial cortical defect

The right tibia was prepared as previously described (Kim et al., 2007; Liu et al., 2018). Briefly, a 0.78 mm monocortical circular defect was made using a #68 sterilized drill bit attached to a Dremel tool (Bosch Tool Group, Model 395). It was centered on the anterior medial cortex of the tibia and was located 4.3 mm from the tibial plateau. Following drilling, the cortical defect was irrigated with sterile saline with the wound closed using 5-0 nylon sutures (McKesson, #1034511). Mice were allowed to heal for 7 days post-injury (PID 7), when the woven bone response, formed *via* intramembranous ossification, encompasses the entire localized marrow space (Supplementary Figure S1) (Uthgenannt et al., 2007; Martinez et al., 2010).

### Frozen histology

Injured and contralateral uninjured limbs were harvested at previously mentioned timepoints (transverse femoral fracture–PID14, ulnar stress fracture–PID10, tibial cortical defect–PID7) and immediately fixed in 4% paraformaldehyde (Electron Microscopy Sciences; #15710) for 24 h. A small subset of transverse femoral fracture femurs (injured + contralateral) were also harvested at days 5 (pulse TMX dosing strategy) and day 7 (continuous TMX dosing strategy) to investigate Osx^+^ and DMP1^+^ lineage cells in the rapidly expanding periosteum and mesenchyme before robust woven bone formation. All specimens underwent standard decalcification for 14 days (14% EDTA, pH 7.0) and subsequent tissue processing (30% sucrose infiltration) followed by embedding and freezing in O.C.T. Compound (Tissue-Tek^®^; #25608-930). Sections were cut longitudinally at a thickness of 5 µm using the Leica CryoJane Tape-Transfer System and stored at −80°C until use.

### Imaging and TdTomato quantification

Slides were rehydrated in deionized water, counterstained using DAPI (Sigma-Aldrich, #D9542, 1:1,000 in DiH_2_0), and mounted with Fluoromount aqueous mounting media (Thermo Fisher Scientific, #00-4958-02). Sections were subsequently imaged under consistent exposure settings for DAPI and TRITC signal at 20–40× magnification by the Nanozoomer Digital Slide Scanning System (Hamamatsu, S360 System). Images containing both channels (DAPI; TdTomato) were exported using NDP. viewer2 (Hamamatsu, #U12388-01) software with consistent image settings (Contrast = 200%; *γ* = 1.8).

#### Contralateral uninjured femur analysis

40X images were randomly taken from each cortical diaphyseal quadrant (ROIs: anterior-proximal; anterior-distal; posterior-proximal; posterior-distal) from uninjured D7 and D14 continuous TMX and vehicle mice from each Cre_ERT2 line. We did not see any differences in TdTomato expression (Cre activation) between uninjured D7 and D14 images. Images were blinded and manually counted for TdTomato positive (TdT^+^) osteocytes, periosteal labeled surface and endosteal labeled surface using the FIJI (Schindelin et al., 2012) ROI manager and cell counter plug-in. TdTomato positive (TdT^+^) cells were normalized to total number of osteocytes or endosteal/periosteal bone surface length for their respective indices. Indices for all four cortical ROIs were averaged on each specimen for final data statistical analysis. 20x images from the femoral mid-diaphysis and distal femoral growth plate were also captured to qualitatively determine relative targeting of skeletal muscle, marrow cells, and chondrocytes based on cellular morphology and anatomical location (Supplementary Figure S2).

#### PID14 femur fracture analysis

*Woven Bone (Intramembranous Region):* Any tissue between the skeletal muscle and cortical bone was considered callus tissue. 40X images were randomly taken from two woven bone regions in the callus (∼2.5–3 mm peripheral to the fracture site), one on the anterior side of the bone and the other on the posterior side. Images were blinded and manually counted for TdTomato positive (TdT^+^) osteocytes within woven bone (Wo.B). Osteocytes were counted as any cell within the woven bone (Wo.B) tissue, excluding the bone surface and adjacent marrow spaces (marked by clusters of overlapping cells). The multi-layered outline of cells encompassing the perimeter of the woven bone (i.e. expanded periosteal perimeter) was also manually counted for TdT^+^ cells using the FIJI (Schindelin et al., 2012) ROI manager and cell counter plug-in. Both indices were normalized to total Wo. B osteocytes and callus perimeter length, respectively. TdT^+^ cellular area was also computed automatically by FIJI as per previous methods and normalized to total callus area (Wang et al., 2019; Shihan et al., 2021). In brief, TdT^+^ cell area was counted automatically by collecting data only on the red channel (split channel function), thresholding to make the image binary (threshold 190), and calculating the thresholded area (particle analysis–no restrictions on size/circularity).

*Cartilage (Endochondral Region):* 40X images were randomly taken from two cartilage regions anterior and posterior to the fracture site away from the mineralizing woven bone front. TdT^+^ cartilage cellular area was also computed automatically by FIJI as per exact methods listed for the woven bone region and normalized to total cartilage area. TdT^+^ cartilage cells per total cartilage cells were calculated for the same images by splitting the red and blue channels, and using particle analysis to automatically count the ratio of TdT^+^ to DAPI^+^ cells. In brief, TdT^+^ cells were counted by binary thresholding (threshold 190), discretizing overlapping cells by watershed analysis, and running particle analysis (size: 20–200 microns; circularity: 0.2-1.0). DAPI^+^ cells were counted by binary thresholding (threshold 150), discretizing overlapping cells by watershed analysis, and running particle analysis (size: 20–200 microns; circularity: 0.2-1).

#### PID10 stress fracture analysis

To complement the woven bone analysis for femoral fracture mice, the periosteal stress fracture callus was also analyzed. For this, 10X images were taken that were centered at the stress fracture crack line of the compressive region of the callus (this ROI encompassed the majority of the callus). Images were blinded and manually counted for TdT^+^ positive osteocytes within woven bone (TdT^+^ Wo.B Osteocytes) and TdT^+^ callus perimeter similar to methods used for the femoral woven bone analysis. TdT^+^ Wo. B cells were calculated for the same images by splitting the red and blue channels and using particle analysis to automatically count the ratio of TdT^+^ to DAPI^+^ cells within the 10X callus region, regardless of location. This cell population included the total number of TdT^+^ woven bone lining cells, woven bone marrow cells and osteocytes. In brief, TdT^+^ cells were counted by binary thresholding (threshold 150), discretizing overlapping cells by watershed analysis, and running particle analysis (size: 20–200 microns; circularity: 0.2-1). DAPI^+^ cells were counted by binary thresholding (threshold 20), discretizing overlapping cells by watershed analysis, and running particle analysis (size: 20–200 microns; circularity: 0.2-1).

#### PID7 tibial cortical defect analysis

To determine if contributions of pre-existing Osx^+^ and DMP1^+^ lineage cells differ following marrow-derived intramembranous bone repair, a small number of mice were given cortical defect injuries following pulse TMX regimens. (Cortical defect experiments were not performed under the continuous TMX protocol.) 10X images were taken centered at the PID7 cortical defect site around the anterior medial surface of the tibia and used to investigate TdT^+^ cells expression within the intramedullary woven bone.

## Statistics

Quantitative outcomes of TdTomato cellular expression per tissue area (woven bone or cartilage), per perimeter (callus or bone), or per cell number were analyzed within each inducible Cre line (Osx or DMP1). Due to the smaller sample sizes used (*n* = 2-4), data normality was first assessed by Q-Q plots and assumed to be normal if not deviating significantly from a straight diagonal line. Depending on outcome, data was compared by unpaired *t*-test or ANOVA (normally distributed) or Mann-Whitney or Kruskal–Wallis to test for the significant effects of tamoxifen dosing (continuous, pulse, vehicle) in GraphPad Prism Pro Version 9 (La Jolla, CA). The type of statistical test for each figure is noted in the legend. Direct statistical comparisons between Cre lines were avoided due to potential confounding technical differences in Ai9 recombination efficiency between Osx Cre_ERT2 and DMP1 Cre_ERT2 constructs, which may not reflect accurate changes in biology. Mouse sex was not tested as an independent variable because our study wasn’t adequately powered to compute male and female differences (so they were pooled for analysis). For added clarity, data points from male and female mice are represented on graphs as diamonds and circles, respectively as noted in each figure’s caption. Post-hoc Tukey’s (parametric - ANOVA) or Dunn’s (non-parametric - Kruskal–Wallis) were used to determine significance differences between individual groups after accounting for multiple comparisons corrections. Statistical significance was defined as *p* < 0.05 and trending values were denoted as *p* < 0.10. Data are presented as mean ± SD with individual sample sizes for each outcome denoted as data points in each graph and in the figure caption.

## Results

### Inducible Osx Cre_ERT2 under continuous dosing targets a higher percentage of femoral cortical bone cells in uninjured bones compared to inducible DMP1 Cre_ERT2

**FIGURE 3:**
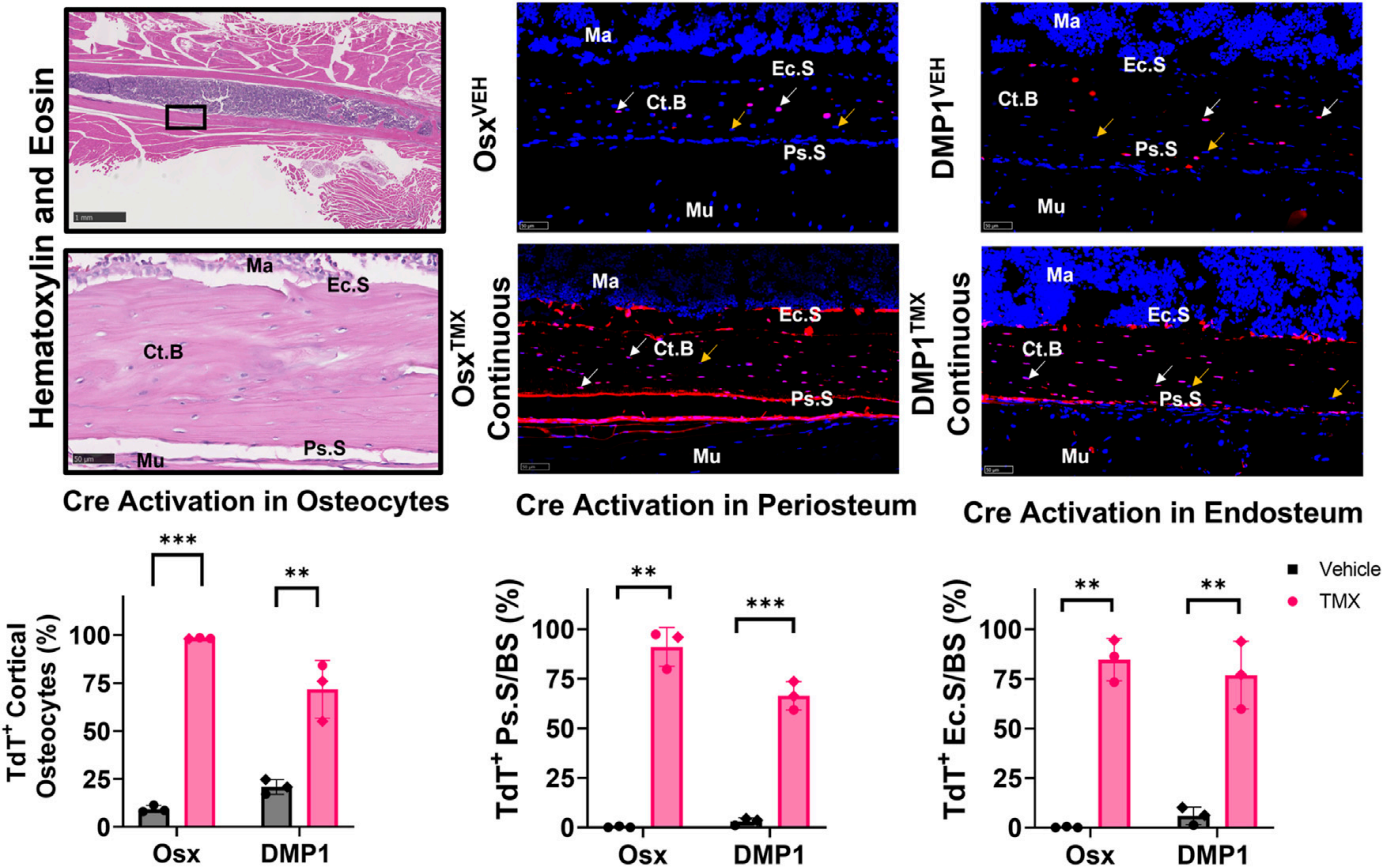
Osx Cre_ERT2 has greater diaphyseal cell targeting than DMP1 Cre_ERT2 in uninjured femurs (osteocytes and bone lining cells). 2.5X (scale bar 1 mm) and 40X images (scale bar 50 µm) were randomly taken from each cortical diaphyseal quadrant (Black ROIs) and used for quantification of Cre specificity from uninjured D7 and D14 continuous TMX and vehicle femurs from each Cre_ERT2 line. TdTomato positive (TdT^+^) osteocytes (white arrow) were normalized to total number of osteocytes (TdT^+^ and TdT^−^ cells - orange arrow). TdTomato positive endosteal (TdT^+^ Ec. S) and periosteal bone surface (TdT^+^ Ps. S) were normalized to total bone surface length (BS). Data presented as mean ± SD with *n* = 3 per group. Mouse sex of each data point is represented by shape (circle–female; diamond–male). Effects between continuous and vehicle dosing within each inducible Cre line were compared by Unpaired *t*-test *p < 0.05; **p < 0.005; ***p < 0.0005 Abbreviations: Ma = Marrow; Ct. B = Cortical bone; Mu = Skeletal muscle.

Uninjured contralateral femurs were first assessed for TdT^+^ cells in intracortical osteocytes and bone surfaces at the mid-diaphysis following continuous TMX administration for 4 weeks. Overall, Osx^TMX^;Continuous femurs showed greater targeting of cells compared to DMP1^TMX^;Continuous femurs in each bone component analyzed (Figure 3). For example, Osx^TMX^;Continuous femurs had 98% of osteocytes labeled TdT^+^ compared to 72% in DMP1^TMX^;Continuous femurs. In addition, Osx^TMX^;Continuous femurs had 91% and 85% of the periosteal and endosteal surfaces labeled, whereas DMP1^TMX^; Continuous femurs had 66% and 77% of the periosteal and endosteal surfaces labeled, respectively (Figure 3). The majority of TdT^+^ labeling was attributed to tamoxifen induction as expected, as both Osx^TMX^;Continuous and DMP1^TMX^;Continuous femurs had significantly increased TdT^+^ labeling in all investigated cortical compartments *versus* respective vehicle-treated controls (*p* < 0.05). In the absence of TMX, the periosteal surface and endosteal surface had negligible non-inducible recombination (“leakiness”) in either Cre_ERT2 line, however leakiness was readily apparent in intracortical osteocytes. For example, 9.4% of osteocytes were TdT^+^ in Osx^VEH^ femurs while in DMP1^VEH^ femurs 21% of osteocytes were TdT^+^ (Figure 3).

Qualitative assessment of TdT^+^ cell labeling outside the cortical diaphyseal bone in the marrow, skeletal muscle and primary spongiosa was also performed (Supplementary Figure S2). Osx^TMX^;Continuous and DMP1^TMX^;Continuous femurs both showed minimal TdT^+^ expression in marrow cells (Supplementary Figure S2; Panel 1). Notably, both DMP1^TMX^; Continuous and DMP1^VEH^ femurs showed robust TdT^+^ expression in skeletal muscle cells, indicative of non-inducible recombination at this site (Supplementary Figure S2; Panel 2). Looking at the distal femoral growth plate, a place undergoing endochondral ossification similar to the fracture callus, Osx^TMX^;Continuous femurs showed greater targeting of growth plate chondrocytes (white arrows) and trabecular bone within the primary spongiosa compared to DMP1^TMX^;Continuous femurs (Supplementary Figure S2; Panel 3). In summary, these results demonstrated that DMP1^VEH^ femurs had greater non-inducible TdTomato expression and hence leakiness in multiple tissue compartments, notably intracortical osteocytes and skeletal muscle, compared to Osx^VEH^. However following a 4-week period of TMX dosing, Osx Cre_ERT2 caused Cre activation in a greater number of bone cells compared to DMP1^TMX^;Continuous mice, such as intracortical osteocytes, periosteal and endosteal lining cells, and growth plate chondrocytes.

### Pulse-chase labeling reveals that pre-existing Osx^+^ but not DMP1^+^ lineage cells and their progeny give rise to most intramembranous woven bone osteocytes following femoral fracture

**FIGURE 4:**
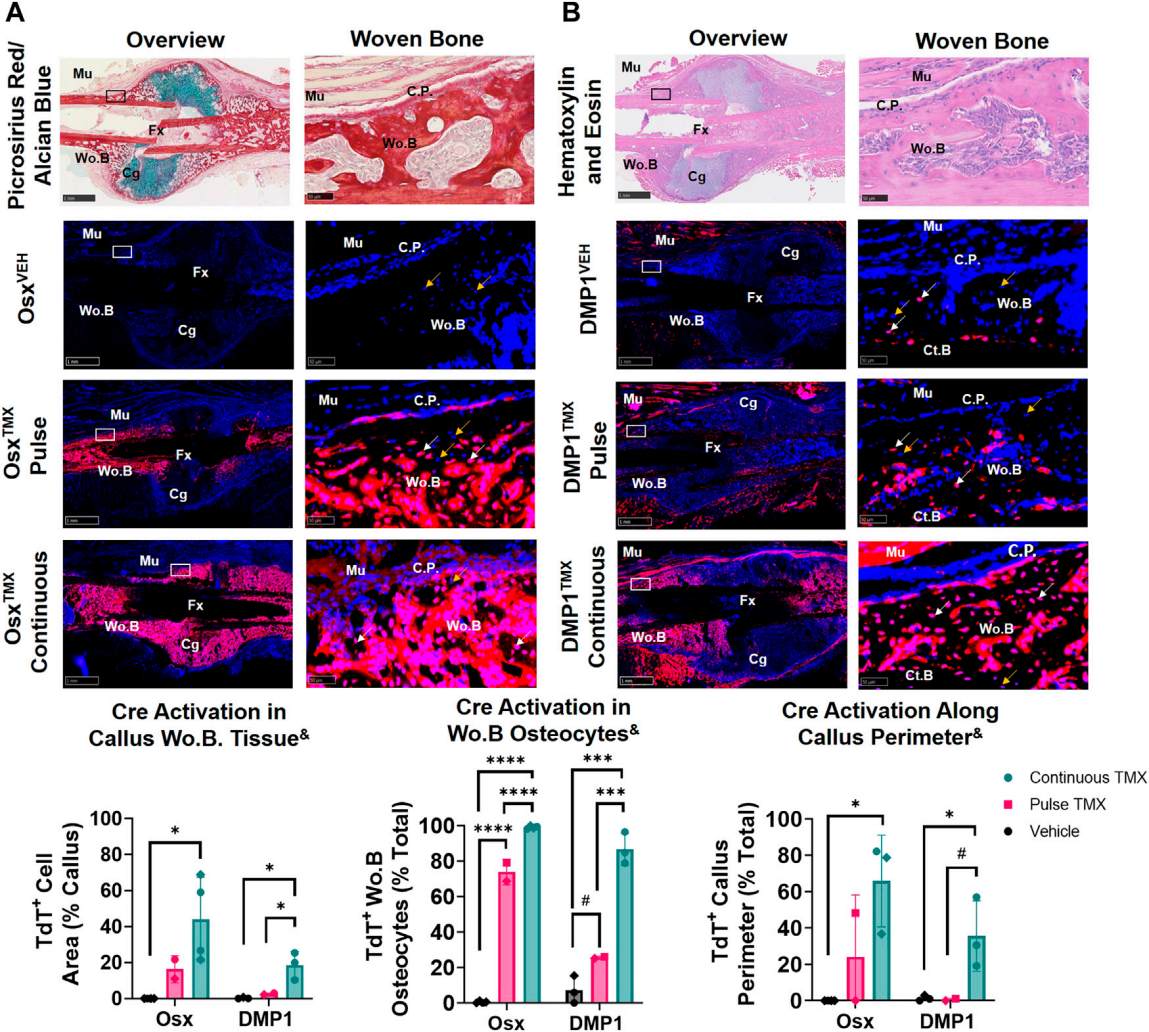
Pre-existing Osx^+^ (panel **(A)**) and DMP1^+^ (panel **(B)**) lineage cells contribute to varying extents of woven bone osteocytes during transverse fracture healing (intramembranous region). 2.5X (Overview; scale bar 1 mm) and 40X images (woven bone - scale bar 50 μm; black/white ROI boxes) were randomly taken and averaged from two regions of interest at the callus periphery >2 mm from PID14 fracture site (Fx) known to contain predominantly woven bone (Wo.B). TdTomato positive (TdT^+^) callus area were calculated from automated thresholding for TdT^+^ area between cortical bone (Ct.B) and skeletal muscle (Mu). Wo.B TdTomato positive (TdT^+^) osteocytes (white arrow) were normalized to total number of osteocytes (TdT^+^ and TdT^−^ cells-orange arrow). TdT^+^ periosteal callus perimeter was normalized to total callus perimeter length (C.P.). Data presented as mean ± SD with *n* = 2-4 per group. Mouse sex of each data point is represented by shape (circle–female; diamond–male). ^&^ Significant Tamoxifen Effect by 1-WAY ANOVA. ^#^ *p* < 0.10; *p < 0.05; ***p < 0.0005; ****p < 0.00005 Significantly Different by Tukey Post-Hoc.

The callus from the fractured femurs was next analyzed for TdTomato expression in woven bone regions at the callus periphery, known to primarily undergo intramembranous ossification, and revealed a large contribution of pre-exisiting Osx^+^ but not DMP1^+^ lineage cells. With pulse dosing, Osx^TMX^;Pulse callus had noticeably increased TdT^+^ stained woven bone area compared to Osx^VEH^ control (16% vs. 0.02%; Figure 4A) but still nearly 3-fold less staining less than Osx^TMX^;Continuous calluses (16% vs. 44%; Figure 4A). Notably, TdT^+^ osteocytes were significantly more abundant in Osx^TMX^;Pulse calluses (74%) than Osx^VEH^ controls (0.47%) although less abundant than Osx^TMX^; Continuous femurs (99%, *p* < 0.05; Figure 4A). Lastly, Osx^TMX^; Pulse femurs also had more TdT^+^ labeled cells lining the perimeter of the intramembranous woven bone callus compared to Osx^VEH^ (24% vs 0%) but this only reached signficance *versus* vehicle in the Osx^TMX^; Continuous group (66% vs 0%, *p* < 0.05; Figure 4A). Taken together, these results indicate that pre-existing Osx^+^ lineage cells and their progeny (identified by pulse-chase labeling) make up about a fifth of intramembranous callus tissue (16%), which is two to three-fold less than the amount labeled by continuous dosing (44%), which captures both pre-existing and newly differentiated Osx^+^ lineage cells and their progeny. Notably, the majority of intramembranous woven bone osteocytes are derived from pre-existing Osx^+^ lineage cells and their progeny (74%).

In contrast, DMP1^TMX^; Pulse calluses demonstrated minimal increases in TdT^+^ woven bone area that was not significantly different from DMP1^VEH^ controls (2.6% vs. 0.3%; Figure 4B). Moreover, DMP1^TMX^; Pulse calluses had significantly less TdT^+^ intramembranous area compared to DMP1^TMX^;Continuous Calluses (2.6% vs 19%; *p* < 0.05; Figure 4B). Differential TdT^+^ labeling between DMP1^TMX;^ Continuous and DMP1 ^TMX;^ Pulse was even more apparent in woven bone intracortical osteocytes and the callus periphery. For instance, DMP1^TMX^; Pulse intramembranous calluses showed a trending but non-significant increase in TdT^+^ osteocytes compared to DMP1^VEH^ controls (26% vs. 7.2%, *p* < 0.10; Figure 4B) but was signficantly less compared to DMP1^TMX^;Continuous calluses (26% vs. 87%, *p* < 0.05; Figure 4B). In addition, only DMP1^TMX^;Continuous calluses had signficantly more TdT^+^ cells lining the periphery of the intramembranous woven bone compared to DMP1^VEH^ (36% vs. 1.3%, *p* < 0.05; Figure 4B). These DMP1^+^ lineage results indicate that pre-existing DMP1^+^ lineage cells contribute minimally to callus formation and only become a small fraction of the total DMP1^+^ cell lineage population (pre-existing and newly-derived). These pre-existing DMP1 lineage cells only contribute to the initial woven bone osteoblasts and osteocytes (marked by proximity to original cortical bone) during fracture healing.

Comparing Osx and DMP1 Cre_ERT2 models, it appears that most osteocytes within woven bone come from pre-existing Osx^+^ lineage cells and their progeny and will acquire DMP1^+^ expression as evidenced by the similar labeling of osteocytes between Osx^TMX^; Pulse and DMP1^TMX^; Continuous calluses (74% *versus* 87%). This is further supported when looking at early timepoints of fracture healing such as PID5 and PID7 in pulsed and continuous fracture calluses, respectively (Supplementary Figures S3, S4). For example, by PID5 a greater extent (i.e. longitudinal length) of the expanded periosteum is labeled by pre-existing Osx^+^ lineage cells than DMP1^+^ lineage cells (Supplementary Figure S3) resulting in a greater proportion of pre-existing and newly-derived Osx^+^ lineage cells and their progeny compared to DMP1^+^ lineage cells and their progeny within woven bone tissue at PID7 (Supplementary Figure S4). Overall, our results indicate that pre-existing Osx^+^ lineage cells and their progeny, but not DMP1^+^ lineage cells (and their progeny), contribute to early woven bone formation in the fracture callus both by lining new woven bone surfaces and becoming embedded osteocytes.

### Newly-derived but not pre-existing Osx^+^ and DMP1^+^ lineage cells and their progeny make up cartilage callus following femoral fracture

**FIGURE 5:**
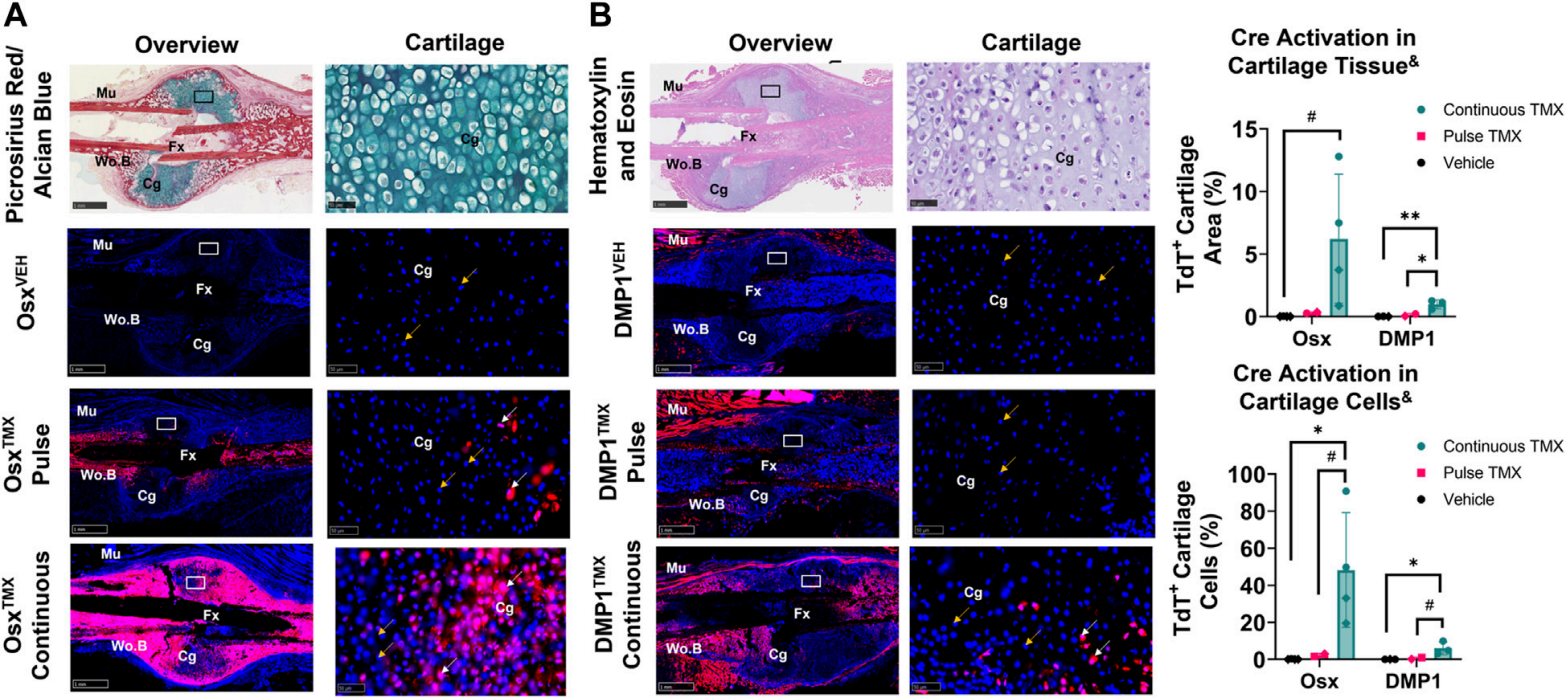
Newly-derived but not pre-existing Osx^+^ (panel **(A)**) and DMP1^+^ (panel **(B)**) lineage cells and their progeny make up cartilage callus following femoral fracture (endochondral region). 2.5X (Overview; scale bar 1 mm) and 40X images (cartilage - scale bar 50 μm; black/white ROI boxes) were randomly taken and averaged from two regions of interest centered in cartilage regions adjacent to the PID14 fracture site (Fx). TdTomato positive callus area were calculated from automated thresholding for TdT^+^ area within each field of view. Cartilage TdTomato positive cells (TdT^+^ Cg cells - white arrow) were thresholded, counted and normalized to total number of DAPI^+^ chondrocytes (TdT^+^ and TdT^−^ cells - orange arrow). Data presented as mean ± SD with *n* = 2–4 per group. Mouse sex of each data point is represented by shape (circle–female; diamond–male). ^&^ Significant Tamoxifen Effect by 1-WAY ANOVA. #*p* < 0.10, *p < 0.05; **p < 0.005; Significantly Different by Tukey Post-Hoc.

TdTomato expression in multiple cartilage regions immediately adjacent to the femoral fracture site was averaged to evaluate the role of pre-existing *versus* newly-derived Osx^+^ and DMP1^+^ lineage cells and their progeny in endochondral ossification at 14 days post-fracture. Overall, we saw little evidence of pre-existing Osx^+^ or DMP1^+^ lineage cells contributing to cartilage formation. For example, with pulse dosing, Osx^TMX^; Pulse calluses had non-signficant TdT^+^ stained cartilage callus tissue (0.3% *versus* 0.0%) and cartilage cells (2.3% *versus* 0.1%) compared to Osx^VEH^ control (Figure 5A). However, with continuous tamoxifen dosing there were trending increases in Osx^TXM^;Continuous TdT^+^ stained callus tissue (6.2 *versus* 0.3%) and cells (48% *versus* 2.3%; *p* < 0.10) compared to Osx^VEH^ control (Figure 5A) indicating the majority of Osx^+^ lineage cartilage cells are newly-derived following fracture.

Similarly, with pulse dosing, DMP1^TMX^; Pulse calluses had minimal cartilage callus area (0.1% *versus* 0.0%) and cartilage cells (0.7% *versus* 0.0%) targeted compared to DMP1^VEH^ but this was significantly enhanced with continuous tamoxifen expression (Figure 5B). While continuous TMX dosing resulted in significant cartilage labeling compared to vehicle controls in both Cre lines, Osx^TMX^ Continuous femurs, on average, targeted approximately 10-fold more chondrocytes compared to DMP1^TMX^ Continuous femurs (48% *versus* 6.0%). These data indicate that pre-existing Osx^+^ and DMP1^+^ lineage cells and their progeny give rise to minimal chondrocytes in the fracture callus. However, it appears that a large portion of total chondrocytes become Osx^+^ lineage cells once formed in the fracture callus between PID7 and PID14, with an even smaller population of chondrocytes becoming DMP1^+^ lineage cells near sites of endochondral ossification.

### Pre-existing Osx^+^ but not DMP1^+^ lineage cells and their progeny contribute a significant but small portion of periosteal woven bone osteocytes following ulnar stress fracture

**FIGURE 6:**
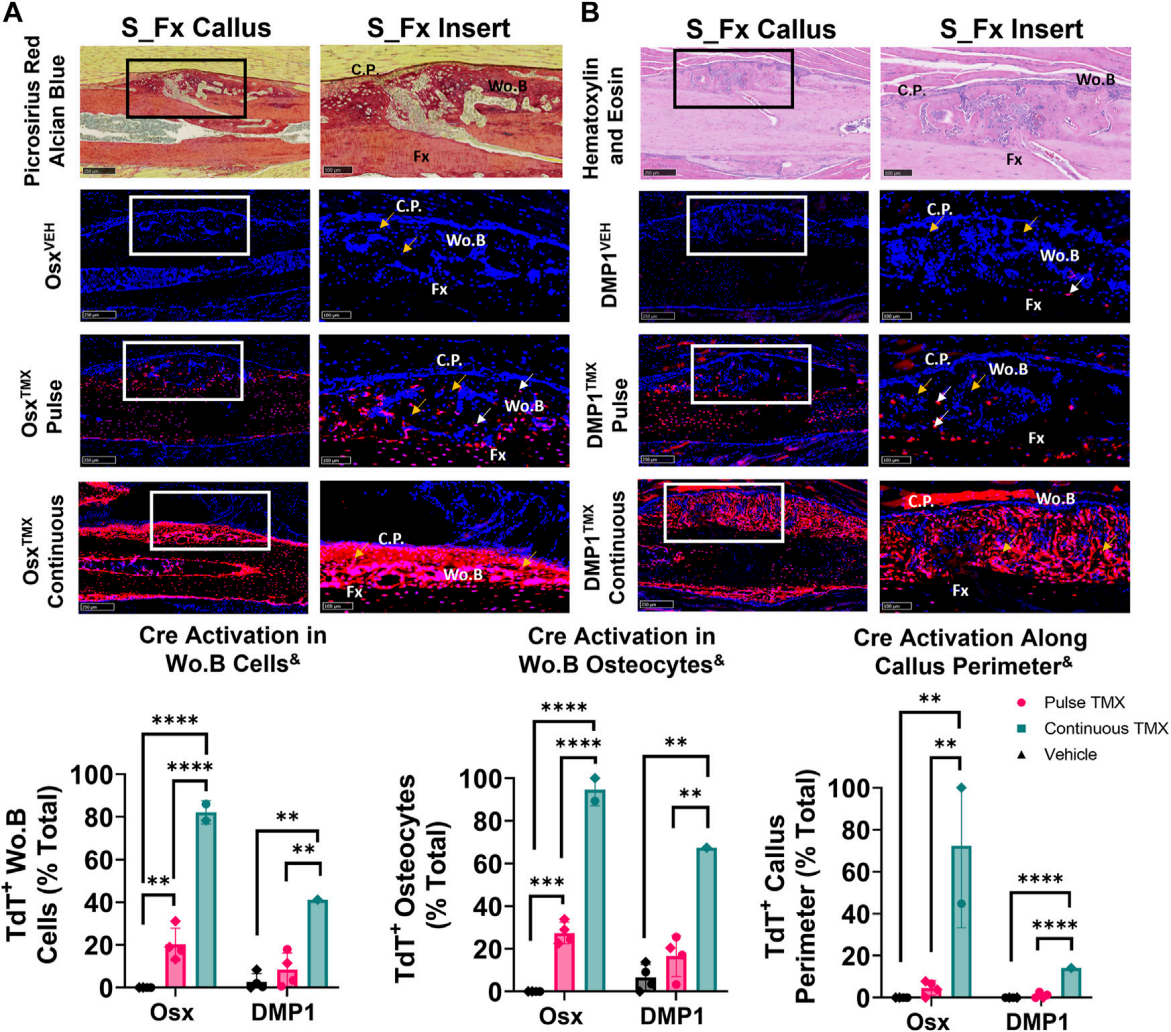
Pre-existing Osx^+^ (panel **(A)**) but not DMP1^+^ (panel **(B)**) lineage cells and their progeny significantly contribute to woven bone cells during stress fracture. 10X and 20X images (S_Fx insert - scale bar 100 µm) centered around the PID10 stress fracture line (Fx). TdTomato positive cells within the callus were thresholded, counted and normalized to total number DAPI^+^ callus cells (TdT^+^ Wo. B Cells). TdTomato positive Wo. B osteocytes (TdT^+^ Wo. B osteocytes - white arrow) were normalized to total number of osteocytes (TdT^+^ and TdT^−^ osteocytes - orange arrow). TdT^+^ periosteal callus perimeter was normalized to total callus perimeter length (C.P.). Data presented as mean ± SD with *n* = 1–4 per group. Mouse sex of each data point is represented by shape (circle–female; diamond–male). ^&^ Significant Tamoxifen Effect by 1-WAY ANOVA. **p < 0.005; ****p < 0.00005 Significantly Different by Tukey Post-Hoc.

The ulnar stress fracture model was utilized in each Cre_ERT2 line (Osx and DMP1) with pulse and continuous TMX dosing to further assess the role of each osteoblast cell lineages’ contribution to periosteal woven bone intramembranous repair. These results partially mirrored the findings in the intramembranous region of the femoral fracture callus and suggest that pre-existing Osx^+^ but not DMP1^+^ lineage cells and their progeny contribute significantly more to woven bone formation following stress fracture. For example, Osx^TMX^; Pulse stress fracture calluses had signficantly increased TdT^+^ cells within the woven bone regions of the stress fracture callus compared to Osx^VEH^ control (20% *versus* 0%; *p* < 0.05; Figure 6A). However, the overall TdT^+^ cell population was significantly less in Osx^TMX^; Pulse calluses compared to Osx^TMX^; Continuous Calluses (20% vs 82%; *p* < 0.05; Figure 6A). Stratifying TdT^+^ cells based on location, the majority of TdT^+^ cells in Osx^TMX^; Pulse stress fracture calluses were embedded woven bone osteocytes (24% of Wo. B osteocytes TdT^+^; *p* < 0.05 compared to 0% in Osx^VEH^) but not callus peripheral cells in the expanded periosteum (4.6% peripheral cells TdT^+^; *p* > 0.05 compared to 0% Osx^VEH^). With continuous TMX dosing, Osx^TMX;^ Continuous calluses showed a significant elevation in TdT^+^ targeting of woven bone osteocytes (95% of Wo. B osteocytes TdT^+^; *p* < 0.05) and expanded callus periosteum (72% of peripheral cells TdT^+^; *p* < 0.05) compared to Osx^TMX^; Pulse and Osx^VEH^ groups (Figure 6A). These data indicate that pre-existing Osx^+^ lineage cells and their progeny make up a small but significant portion of total Osx^+^ lineage cells in intramembranous callus tissue following stress fracture, mainly in the form of woven bone osteocytes. In addition, the majority of intramembranous callus cells acquire Osx^+^ lineage cell specification after injury.

In contrast, DMP1^TMX^; Pulse stress fracture calluses showed no significant elevation of TdTomato expression in callus cells (including total cells, osteocytes, or callus perimeter) *versus* DMP1^VEH^ controls (Figure 6B). However, with continuous TMX dosing, DMP1^TMX^; Continuous stress fracture calluses had significantly elevated TdTomato expression in the callus (41% cells TdT^+^), osteocytes (67% TdT^+^), and callus perimeter cells (14% TdT^+^) compared to DMP1^TMX;^ Pulse and DMP1^VEH^ controls (Figure 6B; *p* < 0.05). Comparing Cre lines with continuous dosing, Osx^TMX^; Continuous had 2-fold greater TdT^+^ labeling in total stress fracture callus cells *versus* DMP1^TMX^; Continuous callus (82% vs 41%). Moreover, Osx^TMX^; Continuous had approximately 1.5-fold greater osteocyte labeling (95% vs. 67%) and nearly 5-fold greater callus peripheral labeling (72% vs. 14%) compared to DMP1^TMX^; Continuous calluses. Collectively, these data indicate that pre-existing Osx^+^ but not DMP1^+^ lineage cells and their progeny contribute a significant number of cells to stress fracture calluses. In addition, although newly-derived Osx^+^ and DMP1^+^ lineage cells and their progeny make up the majority of cells in the stress fracture callus, newly-derived Osx^+^ lineage cells contribute significantly more than newly-derived DMP1^+^ lineage cells to non-osteocytic populations.

### Pre‐exisiting Osx^+^ but not DMP1^+^ lineage cells and their progeny significantly contribute to cells in the intramedullary woven bone following tibial cortical defect

**FIGURE 7:**
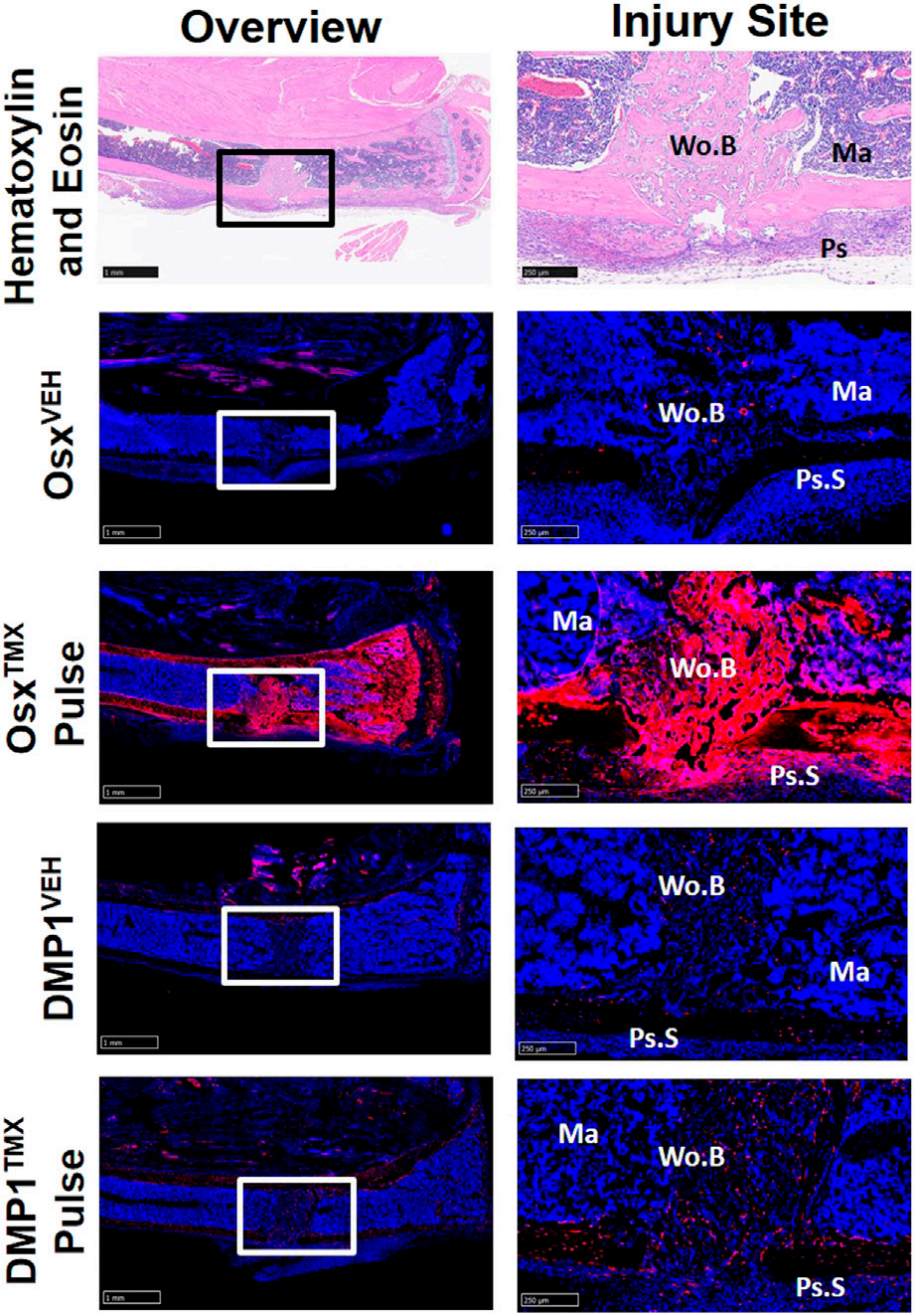
Pre-existing Osx^+^ but not DMP1^+^ Lineage Cells Contribute to Woven Bone Cells During Cortical Defect Healing. 2.5X images (overview - scale bar 1 mm) and 10X images (ROI at injury site - scale bar 250 µm) centered around the PID7 cortical defect. TdTomato positive (TdT^+^) cells were highly visible in woven bone (Wo.B) lining cells, osteocytes, damaged marrow (Ma) and expanded periosteal surface cells (Ps.S) from Osx^TMX^; Pulse mice. DMP1^TMX^; Pulse mice demonstrated sparse TdT^+^ signal in woven bone lining cells, osteocytes, and activated periosteal cells.

To investigate the contribution of Osx^+^ and DMP1^+^ lineage cells and their progeny in another widely used model of bone repair, a tibial cortical defect was created in Osx^TMX^; Pulse and DMP1^TMX;^ Pulse mice. With TMX pulsing, TdT^+^ signal was strongly present in the majority of woven bone cells, including woven bone osteocytes, woven bone lining cells and injured marrow surrounding the defect in Osx^TMX^; Pulse but not DMP1^TMX;^ Pulse mice (Figure 7). Osx^VEH^ and DMP1^VEH^ defects showed minimal non-inducible expression. In all, this suggests that the majority of woven bone cells following cortical defect arises from pre-existing Osx^+^ and their progeny but not pre-existing DMP1^+^ lineage cells and their progeny.

## Discussion

**FIGURE 8:**
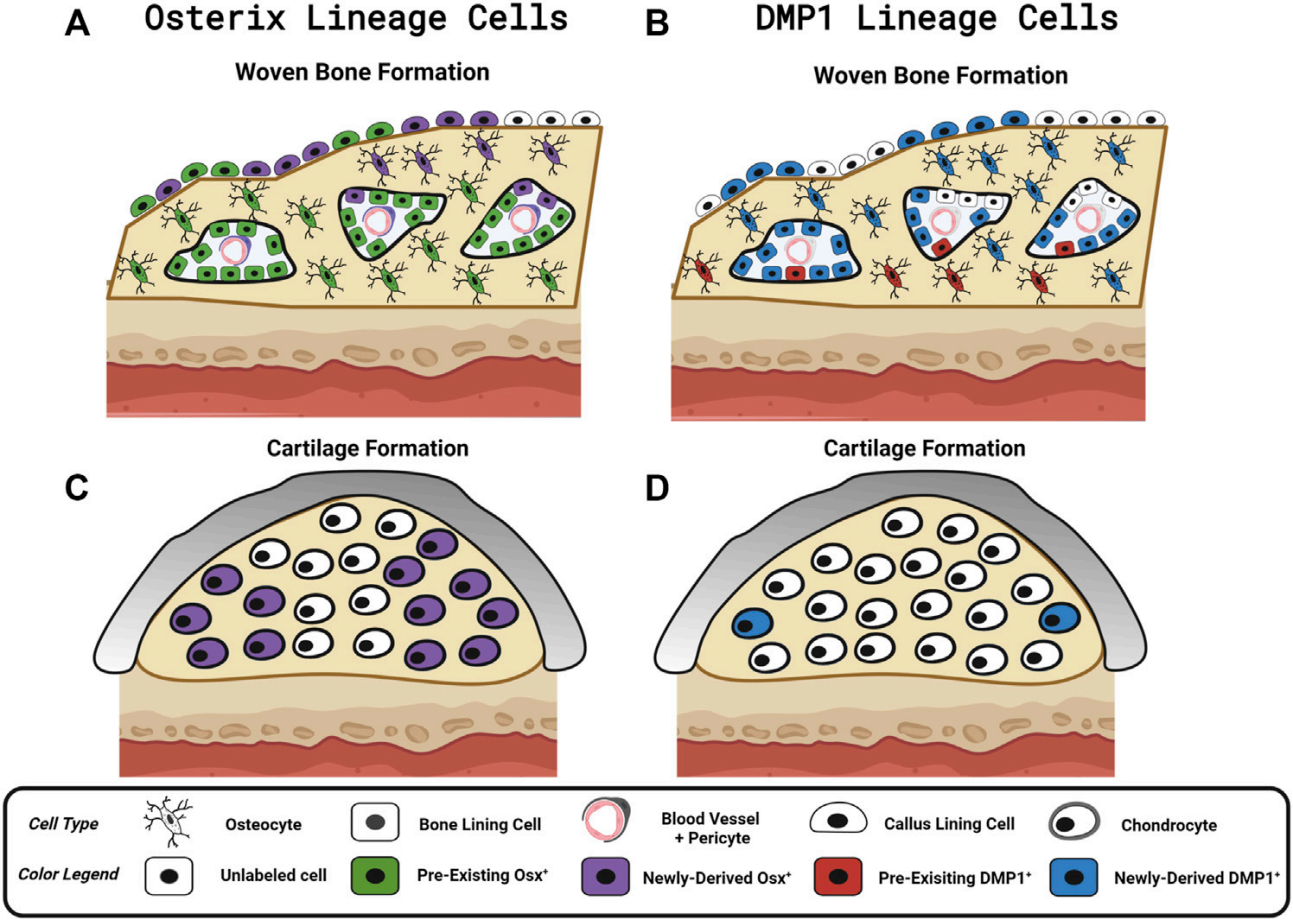
Contribution of Osx and DMP1 Cell Lineages to Bone Repair. Model depicting pre-existing (Pulse TMX) and newly-derived (Continuous TMX) Osx and DMP1 cell lineage contributions to woven bone and cartilage formation in response to bone injury. **(A)** Following full fracture, stress fracture, and cortical defect pre-existing Osx^+^ lineage cells and their progeny (Green) readily become callus lining cells (expanded periosteum), osteocytes and bone lining cells in new woven bone adjacent to the cortical bone surface. Newly-derived Osx^+^ lineage cells (Purple) then make up the rest of the woven bone osteocytes and woven bone lining cells, including the high cellularity marrow spaces, within the intramembranous ossification region. **(B)** In contrast, pre-existing DMP1^+^ lineage cells (Red) sparsely populate new woven bone tissue (i.e. woven bone osteocytes and woven bone lining cells). Although the majority of newly-derived post-fracture woven bone osteocytes and woven bone lining cells go on to express DMP1 (Blue), the high cellularity marrow spaces within woven bone (vasculature and pericytes) are not labeled by DMP1 Cre_ERT2. **(C)** Cells in cartilage regions near the femoral fracture site don’t arise from pre-existing Osx^+^ lineage cells or their progeny. With continuous tamoxifen dosing, a high percentage of chondrocytes are labeled by Osx Cre_ERT2. **(D)** Cells in cartilage regions near the femoral fracture site don’t arise from pre-existing DMP1^+^ lineage cells or their progeny. However, with continuous tamoxifen dosing, a small percentage of chondrocytes express DMP1 (less than Osx^+^ lineage cells). Figure Created in Biorender.

We investigated the contributions of pre-existing *versus* newly-derived Osx^+^ and Dmp1^+^ lineage cells and their progeny to regenerated tissues in three preclinical models of bone injury using inducible Osx Cre_ERT2 Ai9 and DMP1 Cre_ERT2 Ai9 mice. Using two different tamoxifen dosing regimens: 1) pulse-labeling with washout (4 weeks) before injury or 2) biweekly dosing before (2 weeks) and during bone injury healing, we found across injury models (femoral fracture, ulnar stress fracture, tibial cortical defect) that pre-existing Osx^+^ lineage cells and their progeny, but not pre-existing DMP1^+^ lineage cells and their progeny, contributed a significant amount of total TdT^+^ labeled tissue area and cells *versus* respective vehicle controls (Figure 8). These results support our first hypothesis and demonstrate that pre-existing Osx^+^ lineage cells and their progeny but not DMP1^+^ lineage cells and their progeny are a significant source of woven bone forming osteoblasts and osteocytes following bone injury. In addition, continuous tamoxifen administration significantly increased labeling within each inducible Cre line. For example, Osx Cre_ERT2 showing significantly higher targeting of callus tissue with continuous TMX dosing across all scenarios compared to DMP1 Cre_ERT2, supporting our second hypothesis (Figure 8). Importantly, these results suggest that pre-existing Osx^+^ lineage cells and their progeny are likely critical for postnatal injury-induced bone formation, although their contribution varies based on skeletal site and the type of bone injury.

In the femoral fracture callus at day 14 post-injury, the specific Cre model and tamoxifen dosing regimen led to differential targeting of cells based on callus region. Comparing pulse to continuous dosing allowed us to see the maximum contribution of Osx^+^ or DMP1^+^ lineage cells and their progeny to bone healing (pre-existing and newly-derived) within each Cre line. Using this methodology, we saw when comparing Osx^TMX^; Pulse to Osx ^TMX^; Continuous, that the majority of Osx^+^ lineage osteocytes (74% *versus* 99%) and some callus border cells (24% *versus* 66%) and virtually no chondrocytes (2.3% *versus* 48%) were derived from pre-existing Osx^+^ lineage cells and their progeny (Figure 4A and Figure 5A). Comparing DMP1^TMX^; Pulse to DMP1^TMX^; Continuous, we saw that out of all DMP1^+^ lineage cells involved in femoral fracture, only a small percent of pre-existing DMP1^+^ lineage cells become osteocytes (26% *versus* 87%), and virtually none become callus border cells (0.5% *versus* 36%) or chondrocytes (0.7% *versus* 6.0%; Figure 4B and Figure 5B). Our DMP1^TMX^; Pulse results are similar to previous results by Root et al., whom utilized the DMP1 CreERT2; Ai9 mouse crossed to the 2.3 kb Col1 Cre thymidine kinase (tk) mouse (Visnjic et al., 2001) during transcortical fracture healing in 8 week old mice (Root et al., 2020). In this study, the authors used ganciclovir administration (GCV) for 16 days prior to fracture to eliminate the proliferating 2.3Col1 tk^+^ osteoblast lineage cells, which overlap significantly with pulse-labeled DMP1^+^ lineage cells (Matic et al., 2016), leaving only transcortical DMP1^+^ lineage cells prior to injury (Root et al., 2020). Tracing of these pre-labeled transcortical DMP1^+^ lineage cells following fracture for 7 days revealed minimal contribution of DMP1^+^ lineage cells to periosteal woven bone, although all DMP1^+^ lineage cells lining woven bone were 2.3Col1 GFP^+^, suggesting that they were bone-forming osteoblasts (Root et al., 2020). Similarly, our data also suggest minimal contributions of pre-existing DMP1^+^ lineage cells (even those that may not be targeted by 2.3Col1 tk) and their progeny to woven bone following transverse fracture.

Looking at Osx and DMP1 CreERT2 lines collectively in our results indicate that pre-existing Osx^+^ osteoprogenitor lineage cells and their progeny at the time of fracture readily become woven bone callus lining cells, woven bone forming osteoblasts and the majority of embedded osteocytes, whereas pre-existing DMP1^+^ lineage cells and their progeny are mainly absent from fracture callus tissues (a few become woven bone lining osteoblasts and osteocytes) (Figure 8). Furthermore, the higher percentage of overall Osx^+^ lineage cells compared to DMP1^+^ lineage cells in intramembranous callus tissue area (44% *versus* 19%) and chondrocytes (48% *versus* 6.0%), but similar overlap in the percentage of osteocytes (99% *versus* 87%) targeted under continuous dosing regimens suggests that Osx Cre_ERT2 targets a wider population of bone cells that eventually go on to become DMP1^+^ lineage concurrent with woven bone formation and matrix embedding (i.e. osteocytogenesis in woven bone) (Figure 8). This wider targeting of osteoblast lineage cells using Osx Cre_ERT2 over DMP1 Cre_ERT2 is supported by previously published works that DMP1 is expressed at the mature osteoblast and osteocyte stages of differentiation during matrix mineralization and osteocyte cell embedding (Maes et al., 2007; Lu et al., 2011; Powell et al., 2011; Kim et al., 2012; Kalajzic et al., 2013; Matic et al., 2016; Shiflett et al., 2019).

Based on the continuous dosing regimen labeling pre-existing and newly-derived cells and their progeny, we also found that cells will acquire Osx^+^ lineage specification and to a lesser degree DMP1^+^ lineage specification within sites of endochondral ossification at day 14 in the femoral fracture callus (Figure 8). A limitation of this work is that we did not use co-staining to better characterize the identity of these newly-derived Osx^+^ or DMP1^+^ lineage cells observed near the cartilage to bone transition zone (e.g., Collagen type II or Collagen X staining). Another limitation is that our study was underpowered to detect differences in these TdT^+^ cell populations between mouse sexes. As emerging data suggests that mouse sex may differentially regulate the response to tamoxifen (Ceasrine et al., 2019) and lead to biological changes in fracture healing (particularly cartilage formation) (Haffner-Luntzer et al., 2021), future research is needed to determine if mouse sex significantly alters Cre specificity during bone healing. The similar trends in Cre specificity seen between males and females in our data suggest that mouse sex effects are subtle compared to the tamoxifen dosing regimen and Cre construct used for inducible cell targeting. Non-etheless, the anatomic location of these newly-derived Osx^+^ and DMP1^+^ lineage cells and their progeny in both mouse sexes, within the chondrocyte transition zone (near vasculature), are in line with other reports showing that Osx^+^ lineage cells labeled continuously during fracture healing can demonstrate a hypertrophic chondrocyte phenotype (labeled by Collagen X) (Hu et al., 2017; Buettmann et al., 2019). In addition, DMP1 mRNA has previously been shown via *in situ* hybridization to be weakly expressed in a small number of hypertrophic chondrocytes in the growth plate (Lu et al., 2011) and during fracture repair (Toyosawa et al., 2004). These results, along with our own, are consistent with the trans-differentiation of chondrocytes to osteoblast lineage cells as proposed by others (Bahney et al., 2015; Hu et al., 2017). Our use of pulse-labeling strategies extends these prior results and indicates that chondrocytes likely do not arise from pre-existing Osx^+^ or DMP1^+^ lineage cells following bone injury. Therefore, researchers studying conditional gene deletion postnatally during transverse fracture repair would minimize targeting of cartilage cells with Osx Cre_ERT2 or DMP1 Cre_ERT2 mice by using a similar pulse dosing strategy. However, our results differ from Mizoguchi et al., which showed that Osx^+^ lineage cells labeled at postnatal day 5 (P5), can become fracture callus chondrocytes following bone injury nearly 15 weeks later (Mizoguchi et al., 2014). Overall, this suggests that there is a critical time-window between birth and 8 weeks postnatally in which pre-existing Osx^+^ osteoprogenitor cells are bipotent *in vivo*.

In order to complement our femoral fracture results, we tested the requirement of pre-existing and newly-derived Osx^+^ and DMP1^+^ lineage cells and their progeny to contribute to stress fracture repair. This model heals predominantly by intramembranous ossification (Martinez et al., 2010) and has not been extensively explored in the literature using Cre reporter mice. Our results largely mirror the woven bone results seen at day 14 of healing in the intramembranous region of the femoral fracture callus, with pre-existing Osx^+^ but not DMP1^+^ lineage cells and their progeny significantly contributing to callus woven bone cells based on changes from each Cre lines respective vehicle controls (Figure 6). These results reinforce that the stress fracture model largely mirrors the intramembranous processes in the femoral fracture model as we previously reported (Wohl et al., 2009). However, what was striking is that the overall percentage of total Osx^+^ lineage Wo.B osteocytes labeled in pulse versus continuous dosing was much lower in the stress fracture (∼20% total Osx^+^ lineage Wo.B osteocytes came from pre-existing Osx^+^ lineage cells) compared to femoral fracture (∼75% total Osx^+^ lineage Wo.B osteocytes came from pre-existing Osx^+^ lineage cells). In contrast, osteocytes expressing DMP1^+^ cell lineage between pulse vs. continuous labeling were relatively unchanged (∼20–25% total DMP1^+^ lineage Wo.B osteocytes came from pre-existing DMP1^+^ lineage cells) between full fracture and stress fracture repair. These findings suggest, that pre-existing Osx^+^ lineage osteoprogenitors and their progeny contribute less to total callus area and cellularity in the less traumatic ulnar stress fracture than the femoral fracture model. The overall result that pre-existing Osx^+^ lineage cells contribute more to callus cells than DMP1^+^ lineage cells with higher degrees of bone damage are consistent with previous reports using anabolic tibial loading at graded force levels (Harris and Silva, 2022), and may potentially reflect the smaller overall cellularity and decreased proliferative processes in stress fracture *versus* full fracture injuries as previously shown (Coates et al., 2019). However, it may also reflect changes in anatomic location (ulna *versus* femur) or slight differences in analysis regions between the two fracture models used in the current study (i.e. majority of callus used to analyze stress fracture vs. callus periphery in transverse fracture).

Despite these differences in pre-existing Osx^+^ lineage cell recruitment, the full fracture and stress fracture model also show some striking similarities in the types of cells targeted between both inducible Cre drivers. For example, in both models, Osx^TMX^; Continuous but not DMP1^TMX^; Continuous labeling results in a strong TdT^+^ signal within woven bone marrow spaces known as sites of progenitor cell and blood vessel invasion (Figure 4 and Figure 6) that support bone healing (Hausman et al., 2001; Lu et al., 2006; Tomlinson et al., 2013). Furthermore, Osx^TMX^; Pulse labeling shows much weaker TdT^+^ signal compared to Osx^TMX^; Continuous calluses at these woven bone marrow sites. Maes et al. has demonstrated previously that osteoblast precursors labeled instantaneously by Osx Cre (but not Collagen 1 Cre), can take on a pericyte-like profile and co-invade woven bone spaces in the fracture callus, thereby supporting angiogenesis and subsequent bone formation (Maes et al., 2010). This concept was further supported by Buettmann et al., where using continuous dosing in Osx Cre_ERT2 VEGFA^fl/fl^ mice led to decreased femoral fracture and stress fracture angiogenesis and subsequent woven bone formation (Buettmann et al., 2019). Our pulse labeling strategy expands upon these results and suggests that pre-existing Osx^+^ lineage cells and their progeny, due to reduced TdT^+^ targeting of Wo.B marrow cells in femoral fracture and ulnar stress fracture, likely do not co-invade with vasculature (neither do more mature DMP1^+^ lineage cells). Thus, if Osx Cre_ERT2 VEGFA^fl/fl^ mice were pulse-dosed with TMX (rather than continuously dosed as previously performed in Buettmann et al., 2019), we hypothesize that femoral fracture and ulnar stress fracture healing would not be impaired.

Lastly, we showed that pre-existing Osx^+^ but not DMP1^+^ lineage cells and their progeny make up a majority of intramedullary woven bone tissue following monocortical defect. In particular, Osx^TMX^; Pulse showed TdT^+^ cells encompassing the majority of woven bone surfaces, osteocytes and even adjacent marrow, whereas these sites were largely void of TdT^+^ expression in DMP1^TMX^; Pulse defects (Figure 7). These results indicate that, at 8 weeks age, pre-existing Osx^+^ lineage cells but not pre-existing DMP1^+^ lineage cells and their progeny significantly contribute to intramedullary bone formation following cortical defect. Although the exact bone compartment contributing to this differential TdTomato expression is unknown, work by Colnot suggests that both endosteal and marrow derived cellular niches act locally to play a large role in monocortical defect healing (Colnot, 2009). Therefore, it is likely that the pre-existing Osx^+^ but not pre-existing DMP1^+^ lineage cells contributing to defect labeling are derived from the endosteum or marrow niche. Although continuous labeling revealed similar endosteal (Figure 3) and minimal marrow (Supplementary Figure S2; Panel 1) targeting in Osx Cre_ERT2 and DMP1 Cre_ERT2 mice, pre-existing lineage cells at these sites were not quantitated in pulse-labeled uninjured specimens, which is a limitation of the current work. Other reports indicate that later pulse labeling (14 days postnatal or after) in Osx Cre_ERT2 and DMP1 Cre_ERT2 labels vascular associated reticular marrow cells and endosteal bone-lining cells that decrease in number over time (Powell et al., 2011; Kim et al., 2012; Park et al., 2012). For example, Matic et al. demonstrated that DMP1^+^ lineage endosteal bone lining cells decrease by 50–75% 3 weeks following tamoxifen induction (Matic et al., 2016). Therefore, it is possible that the differential Osx^+^ and DMP1^+^ lineage cell labeling in the intramedullary woven bone seen in our study is due to a preferential decline in DMP1^+^ over Osx^+^ lineage endosteal cells during the 4 weeks between pulse labeling and the cortical defect creation. Another possibility is that Osx Cre_ERT2 targets a marrow or endosteal lineage cell population with higher regenerative capacity overall compared to DMP1 Cre_ERT2. This differential Cre specificity would be in line with previous reports showing that peri-vascular stromal Osx^+^ lineage cells in the marrow have high regenerative capacity following bone injury (Park et al., 2012; Mizoguchi et al., 2014). Future studies, using dual-labeling strategies, to determine the instantaneous degree of overlap between Osx^+^ and DMP1^+^ lineage cells in various bone compartments, would be particularly informative.

In all, we have shown in the current study that pre-existing postnatal Osx^+^ lineage cells and not pre-existing DMP1^+^ lineage cells and their progeny contribute significantly to cells populating woven bone in multiple widely used preclinical models of bone injury. This study underscores the importance that pre-existing Osx^+^ lineage cells play in bone regeneration, especially for early woven bone formation, and suggest that bone targeting therapies to improve healing might target this particular cellular subset. Furthermore, this work provides a tissue and cellular atlas for inducible Cre targeting using the Osx Cre_ERT2 and DMP1 Cre_ERT2 models during bone healing, thereby providing a framework for researchers using these widely available tools in future studies.

## Data Availability

The original contributions presented in the study are included in the article/Supplementary Material, further inquiries can be directed to the corresponding author.

## References

[B1] Abe T. Fujimori T. (2013). Reporter mouse lines for fluorescence imaging. Dev. Growth Differ. 55 (4), 390–405. 10.1111/dgd.12062 23621623

[B2] An Y. Friedman R. J. ParenT T. Draughn R. A. (1994). Production of a standard closed fracture in the rat tibia. J. Orthop. Trauma 8 (2), 111–115. 10.1097/00005131-199404000-00006 8207566

[B3] Bahney C. S. Hu D. P. Miclau T. Marcucio R. S. (2015). The multifaceted role of the vasculature in endochondral fracture repair. Front. Endocrinol. 6, 4. 10.3389/fendo.2015.00004 PMC4318416 25699016

[B4] Bonafede M. Espindle D. Bower A. G. (2013). The direct and indirect costs of long bone fractures in a working age US population. J. Med. Econ. 16 (1), 169–178. 10.3111/13696998.2012.737391 23035626

[B5] Bonnarens F. Einhorn T. A. (1984). Production of a standard closed fracture in laboratory animal bone. J. Orthop. Res. 2 (1), 97–101. 10.1002/jor.1100020115 6491805

[B6] Buettmann E. G. McKenzie J. A. Migotsky N. Sykes D. A. Hu P. Yoneda S. (2019). VEGFA from early osteoblast lineage cells (Osterix+) is required in mice for fracture healing. J. Bone Min. Res. 34 (9), 1690–1706. 10.1002/jbmr.3755 PMC6744295 31081125

[B7] Ceasrine A. M. Ruiz-Otero N. Lin E. E. Lumelsky D. N. Boehm E. D. Kuruvilla R. (2019). Tamoxifen improves glucose tolerance in a delivery-sex-and strain-dependent manner in mice. Endocrinology 160 (4), 782–790. 10.1210/en.2018-00985 30759201 PMC6424092

[B8] Coates B. McKenzie J. A. Buettmann E. G. Liu X. Gontarz P. M. Zhang B. (2019). Transcriptional profiling of intramembranous and endochondral ossification after fracture in mice. Bone 127, 577–591. 10.1016/j.bone.2019.07.022 31369916 PMC6708791

[B9] Colnot C. (2009). Skeletal cell fate decisions within periosteum and bone marrow during bone regeneration. J. Bone Min. Res. 24 (2), 274–282. 10.1359/jbmr.081003 PMC3276357 18847330

[B10] Einhorn T. A. Gerstenfeld L. C. (2015). Fracture healing: Mechanisms and interventions. Nat. Rev. Rheumatol. 11 (1), 45–54. 10.1038/nrrheum.2014.164 25266456 PMC4464690

[B11] Feil S. Valtcheva N. Feil R. (2009). Inducible Cre mice. Methods Mol. Biol. 530, 343–363. 10.1007/978-1-59745-471-1_18 19266339

[B12] Haffner-Luntzer M. Fischer V. Ignatius A. (2021). Differences in fracture healing between female and male C57bl/6J mice. Front. Physiol. 12, 712494. 10.3389/fphys.2021.712494 34434120 PMC8381649

[B13] Harris T. L. Silva M. J. (2022). Dmp1 lineage cells contribute significantly to periosteal lamellar bone formation induced by mechanical loading but are depleted from the bone surface during rapid bone formation. JBMR Plus 6 (3), e10593. 10.1002/jbm4.10593 35309865 PMC8914163

[B14] Hausman M. R. Schaffler M. B. Majeska R. J. (2001). Prevention of fracture healing in rats by an inhibitor of angiogenesis. Bone 29 (6), 560–564. 10.1016/s8756-3282(01)00608-1 11728927

[B15] Hsieh Y. F. Silva M. J. (2002). *In vivo* fatigue loading of the rat ulna induces both bone formation and resorption and leads to time-related changes in bone mechanical properties and density. J. Orthop. Res. 20 (4), 764–771. 10.1016/S0736-0266(01)00161-9 12168665

[B16] Hu D. P. Ferro F. Yang F. Taylor A. J. Chang W. Miclau T. (2017). Cartilage to bone transformation during fracture healing is coordinated by the invading vasculature and induction of the core pluripotency genes. Development 144 (2), 221–234. 10.1242/dev.130807 28096214 PMC5394763

[B17] Hu K. Olsen B. R. (2016). Osteoblast-derived VEGF regulates osteoblast differentiation and bone formation during bone repair. J. Clin. Invest. 126 (2), 509–526. 10.1172/JCI82585 26731472 PMC4731163

[B18] Julien A. Perrin S. Martinez-Sarra E. Kanagalingam A. Carvalho C. Luka M. (2022). Skeletal stem/progenitor cells in periosteum and skeletal muscle share a common molecular response to bone injury. J. Bone Min. Res. 37 (8), 1545–1561. 10.1002/jbmr.4616 PMC9543664 35652423

[B19] Kalajzic I. Matthews B. G. Torreggiani E. Harris M. A. Divieti Pajevic P. Harris S. E. (2013). *In vitro* and *in vivo* approaches to study osteocyte biology. Bone 54 (2), 296–306. 10.1016/j.bone.2012.09.040 23072918 PMC3566324

[B20] Kim J. B. Leucht P. Lam K. Luppen C. Ten Berge D. Nusse R. (2007). Bone regeneration is regulated by wnt signaling. J. Bone Min. Res. 22 (12), 1913–1923. 10.1359/jbmr.070802 17696762

[B21] Kim S. W. Pajevic P. D. Selig M. Barry K. J. Yang J. Y. Shin C. S. (2012). Intermittent parathyroid hormone administration converts quiescent lining cells to active osteoblasts. J. Bone Min. Res. 27 (10), 2075–2084. 10.1002/jbmr.1665 PMC3529414 22623172

[B22] Li Z. Helms J. A. (2021). Drill hole models to investigate bone repair. Methods Mol. Biol. 2221, 193–204. 10.1007/978-1-0716-0989-7_12 32979205

[B23] Liu C. Carrera R. Flamini V. Kenny L. Cabahug-Zuckerman P. George B. M. (2018). Effects of mechanical loading on cortical defect repair using a novel mechanobiological model of bone healing. Bone 108, 145–155. 10.1016/j.bone.2017.12.027 29305998 PMC8262576

[B24] Lu C. Marcucio R. Miclau T. (2006). Assessing angiogenesis during fracture healing. Iowa Orthop. J. 26, 17–26. 16789443 PMC1888583

[B25] Lu Y. Yuan B. Qin C. Cao Z. Xie Y. Dallas S. L. (2011). The biological function of DMP-1 in osteocyte maturation is mediated by its 57-kDa C-terminal fragment. J. Bone Min. Res. 26 (2), 331–340. 10.1002/jbmr.226 PMC3179348 20734454

[B26] Madisen L. Zwingman T. A. Sunkin S. M. Oh S. W. Zariwala H. A. Gu H. (2010). A robust and high-throughput Cre reporting and characterization system for the whole mouse brain. Nat. Neurosci. 13 (1), 133–140. 10.1038/nn.2467 20023653 PMC2840225

[B27] Maes C. Kobayashi T. Selig M. K. Torrekens S. Roth S. I. Mackem S. (2010). Osteoblast precursors, but not mature osteoblasts, move into developing and fractured bones along with invading blood vessels. Dev. Cell. 19 (2), 329–344. 10.1016/j.devcel.2010.07.010 20708594 PMC3540406

[B28] Maes C. Kobayashi T. Kronenberg H. M. (2007). A novel transgenic mouse model to study the osteoblast lineage *in vivo* . Ann. N. Y. Acad. Sci. 1116, 149–164. 10.1196/annals.1402.060 18083926

[B29] Marsell R. Einhorn T. A. (2011). The biology of fracture healing. Injury 42 (6), 551–555. 10.1016/j.injury.2011.03.031 21489527 PMC3105171

[B30] Martinez M. D. Schmid G. J. McKenzie J. A. Ornitz D. M. Silva M. J. (2010). Healing of non-displaced fractures produced by fatigue loading of the mouse ulna. Bone 46 (6), 1604–1612. 10.1016/j.bone.2010.02.030 20215063 PMC2875275

[B31] Matic I. Matthews B. G. Wang X. Dyment N. A. Worthley D. L. Rowe D. W. (2016). Quiescent bone lining cells are a major source of osteoblasts during adulthood. STEM CELLS 34 (12), 2930–2942. 10.1002/stem.2474 27507737 PMC5450652

[B32] McBride-Gagyi S. H. McKenzie J. A. Buettmann E. G. Gardner M. J. Silva M. J. (2015). Bmp2 conditional knockout in osteoblasts and endothelial cells does not impair bone formation after injury or mechanical loading in adult mice. Bone 81, 533–543. 10.1016/j.bone.2015.09.003 26344756 PMC4640950

[B33] McKenzie J. Smith C. Karuppaiah K. Langberg J. Silva M. J. Ornitz D. M. (2019). Osteocyte death and bone overgrowth in mice lacking fibroblast growth factor receptors 1 and 2 in mature osteoblasts and osteocytes. J. Bone Min. Res. 34 (9), 1660–1675. 10.1002/jbmr.3742 PMC6744314 31206783

[B34] McKenzie J. A. Maschhoff C. Liu X. Migotsky N. Silva M. J. Gardner M. J. (2018). Activation of hedgehog signaling by systemic agonist improves fracture healing in aged mice. J. Orthop. Res. 37, 51–59. 10.1002/jor.24017 29663560 PMC6226344

[B35] Miller G. J. Gerstenfeld L. C. Morgan E. F. (2015). Mechanical microenvironments and protein expression associated with formation of different skeletal tissues during bone healing. Biomech. Model. Mechanobiol. 14 (6), 1239–1253. 10.1007/s10237-015-0670-4 25822264 PMC5608650

[B36] Mizoguchi T. Pinho S. Ahmed J. Kunisaki Y. Hanoun M. Mendelson A. (2014). Osterix marks distinct waves of primitive and definitive stromal progenitors during bone marrow development. Dev. Cell. 29 (3), 340–349. 10.1016/j.devcel.2014.03.013 24823377 PMC4051418

[B37] Park D. Spencer J. A. Koh B. I. Kobayashi T. Fujisaki J. Clemens T. L. (2012). Endogenous bone marrow MSCs are dynamic, fate-restricted participants in bone maintenance and regeneration. Cell. Stem Cell. 10 (3), 259–272. 10.1016/j.stem.2012.02.003 22385654 PMC3652251

[B38] Powell W. F. Jr Barry K. J. Tulum I. Kobayashi T. Harris S. E. Bringhurst F. R. (2011). Targeted ablation of the PTH/PTHrP receptor in osteocytes impairs bone structure and homeostatic calcemic responses. J. Endocrinol. 209 (1), 21–32. 10.1530/JOE-10-0308 21220409 PMC3783949

[B39] Root S. H. Wee N. K. Y. Novak S. Rosen C. J. Baron R. Matthews B. G. (2020). Perivascular osteoprogenitors are associated with transcortical channels of long bones. STEM CELLS 38 (6), 769–781. 10.1002/stem.3159 32053258 PMC7768989

[B40] Schindelin J. Arganda-Carreras I. Frise E. Kaynig V. Longair M. Pietzsch T. (2012). Fiji: An open-source platform for biological-image analysis. Nat. Methods 9 (7), 676–682. 10.1038/nmeth.2019 22743772 PMC3855844

[B41] Seime T. Kolind M. Mikulec K. Summers M. A. Cantrill L. Little D. G. (2015). Inducible cell labeling and lineage tracking during fracture repair. Dev. Growth Differ. 57 (1), 10–23. 10.1111/dgd.12184 25389084

[B42] Serowoky M. A. Arata C. E. Crump J. G. Mariani F. V. (2020). Skeletal stem cells: Insights into maintaining and regenerating the skeleton. Development 147 (5), dev179325. 10.1242/dev.179325 32161063 PMC7075071

[B43] Shiflett L. A. Tiede-Lewis L. M. Xie Y. Lu Y. Ray E. C. Dallas S. L. (2019). Collagen dynamics during the process of osteocyte embedding and mineralization. Front. Cell. Dev. Biol. 7, 178. 10.3389/fcell.2019.00178 31620436 PMC6759523

[B44] Shihan M. H. Novo S. G. Le Marchand S. J. Wang Y. Duncan M. K. (2021). A simple method for quantitating confocal fluorescent images. Biochem. Biophys. Rep. 25, 100916. 10.1016/j.bbrep.2021.100916 33553685 PMC7856428

[B45] Tomlinson R. E. McKenzie J. A. Schmieder A. H. Wohl G. R. Lanza G. M. Silva M. J. (2013). Angiogenesis is required for stress fracture healing in rats. Bone 52 (1), 212–219. 10.1016/j.bone.2012.09.035 23044046 PMC3513671

[B46] Toyosawa S. KaNataNiN. Shintani S. KobataM. YukiM. KishinoM. (2004). Expression of dentin matrix protein 1 (DMP1) during fracture healing. Bone 35 (2), 553–561. 10.1016/j.bone.2004.03.030 15268908

[B47] Uthgenannt B. Kramer M. H. Hwu J. A. Wopenka B. Silva M. J. (2007). Skeletal self-repair: Stress fracture healing by rapid formation and densification of woven bone. J. Bone Min. Res. 22 (10), 1548–1556. 10.1359/jbmr.0070614 PMC3680519 17576168

[B48] Visnjic D. KalajzIc I. Gronowicz G. Aguila H. L. Clark S. H. Lichtler A. C. (2001). Conditional ablation of the osteoblast lineage in Col2.3deltatk transgenic mice. J. Bone Min. Res. 16 (12), 2222–2231. 10.1359/jbmr.2001.16.12.2222 11760835

[B49] Wang K. Le L. Chun B. M. Tiede-Lewis L. M. Shiflett L. A. Prideaux M. (2019). A novel osteogenic cell line that differentiates into GFP-tagged osteocytes and forms mineral with a bone-like lacunocanalicular structure. J. Bone Min. Res. 34 (6), 979–995. 10.1002/jbmr.3720 PMC7350928 30882939

[B50] Wohl G. R. Towler D. A. Silva M. J. (2009). Stress fracture healing: Fatigue loading of the rat ulna induces upregulation in expression of osteogenic and angiogenic genes that mimic the intramembranous portion of fracture repair. Bone 44 (2), 320–330. 10.1016/j.bone.2008.09.010 18950737 PMC2759644

[B51] Woolf A. D. Pfleger B. (2003). Burden of major musculoskeletal conditions. Bull. World Health Organ. 81 (9), 646–656. 14710506 PMC2572542

[B52] Zannit H. M. Silva M. J. (2019). Proliferation and activation of osterix-lineage cells contribute to loading-induced periosteal bone formation in mice. JBMR Plus 3 (11), e10227. 10.1002/jbm4.10227 31768488 PMC6874181

[B53] Zondervan R. L. Vorce M. Servadio N. Hankenson K. D. (2018). Fracture apparatus design and protocol optimization for closed-stabilized fractures in rodents. J. Vis. Exp. 138, 58186. 10.3791/58186 PMC6126799 30176013

