# Supplementary figures and images for: Postnatal Osterix but not DMP1 lineage cells significantly contribute to intramembranous ossification in three preclinical models of bone injury
Source: Front Physiol. 2023 Jan 4;13:1083301. doi: 10.3389/fphys.2022.1083301 (PMC9846510; doi:10.3389/fphys.2022.1083301)

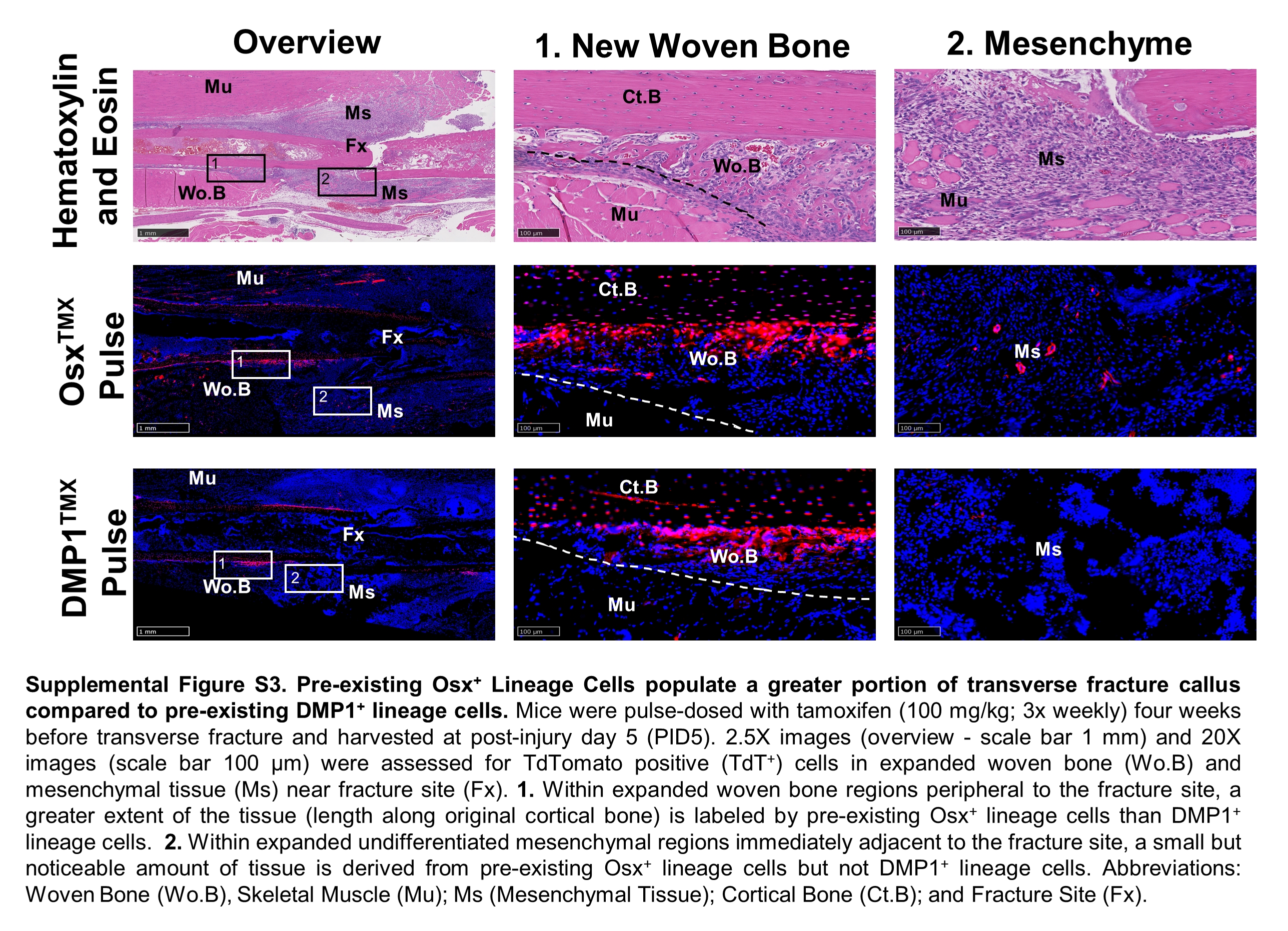

Supplement: Supplementary file 1 [file Image3.jpg]

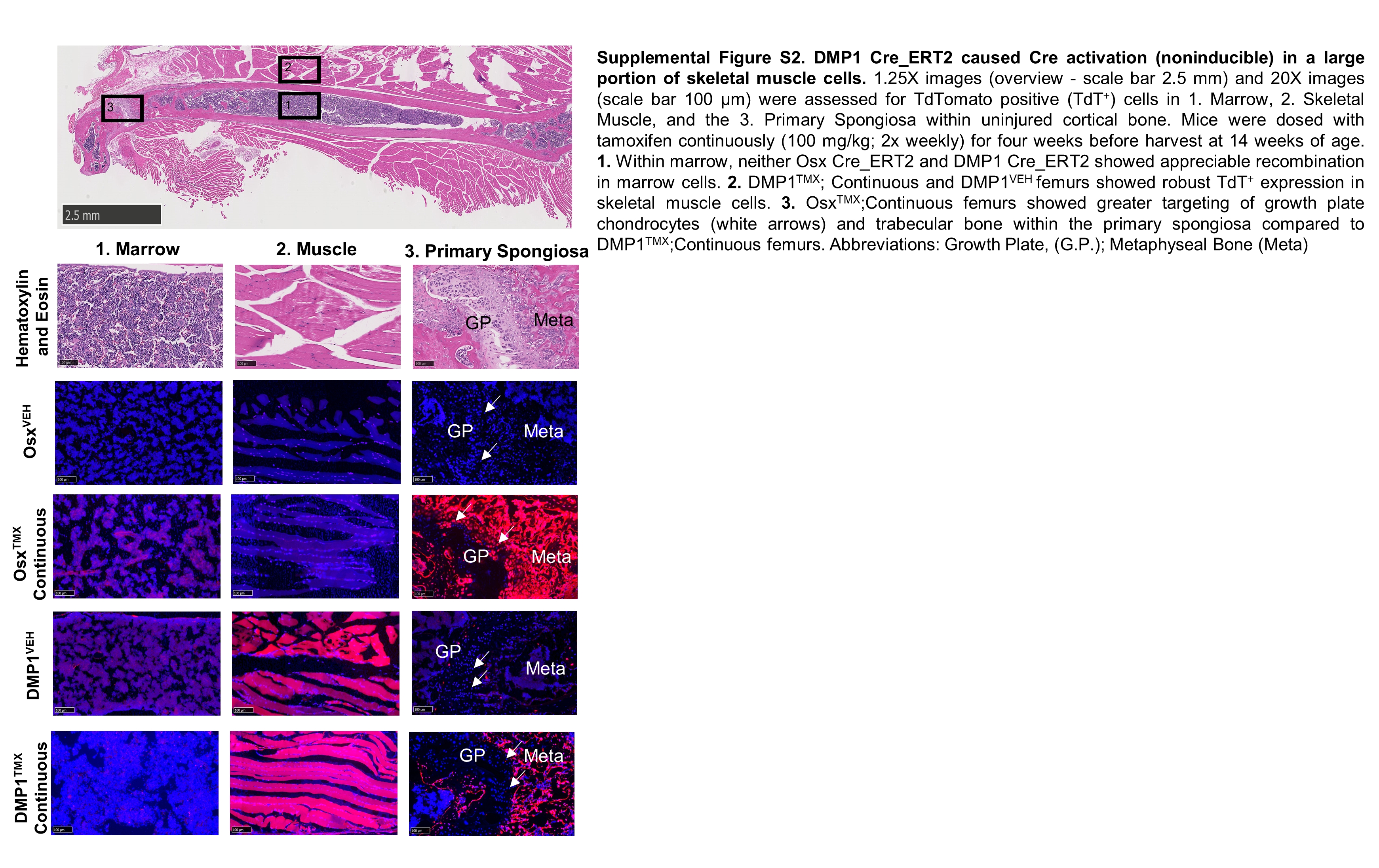

Supplement: Supplementary file 2 [file Image2.jpg]

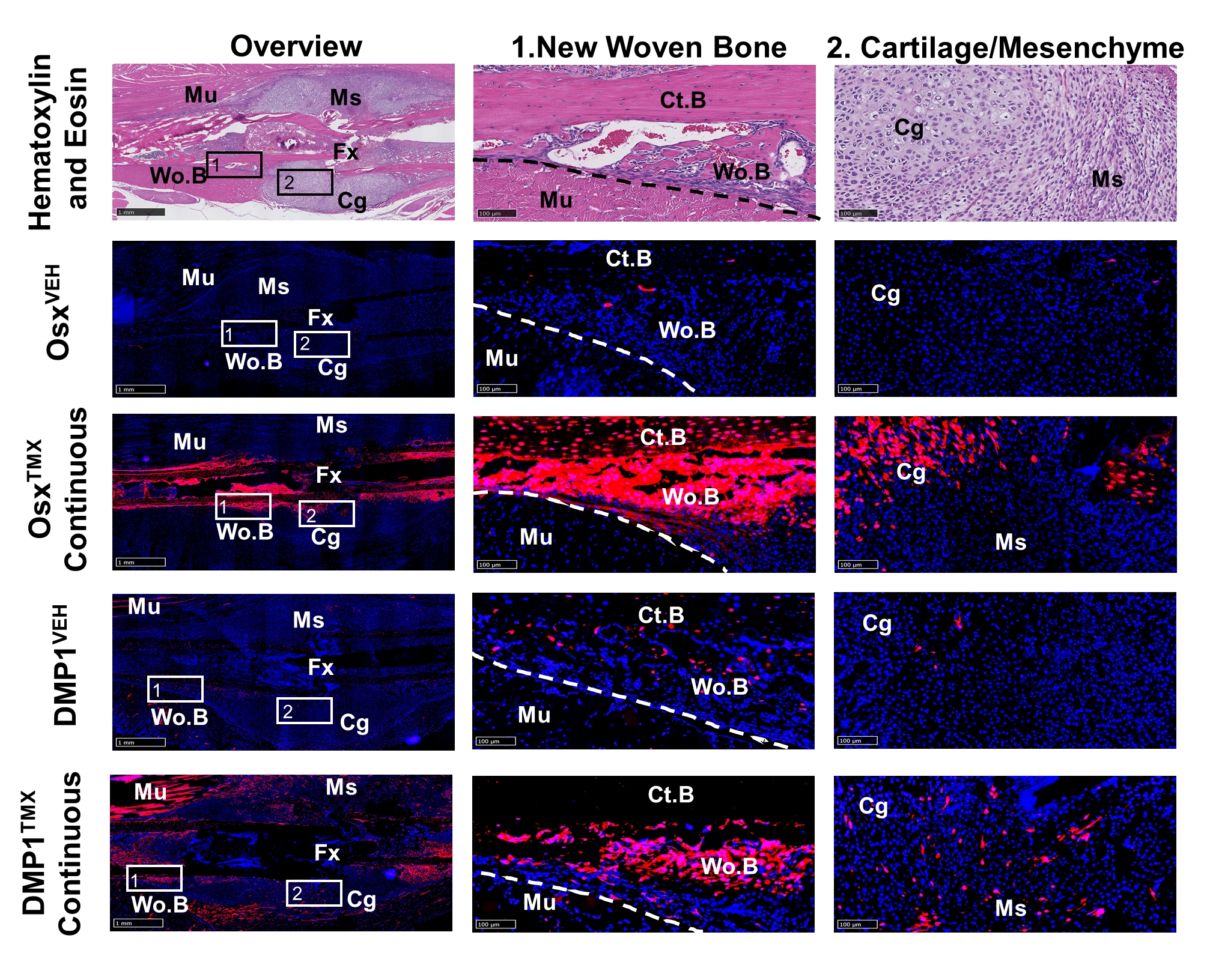

Supplement: Supplementary file 3 [file Image5.JPEG]

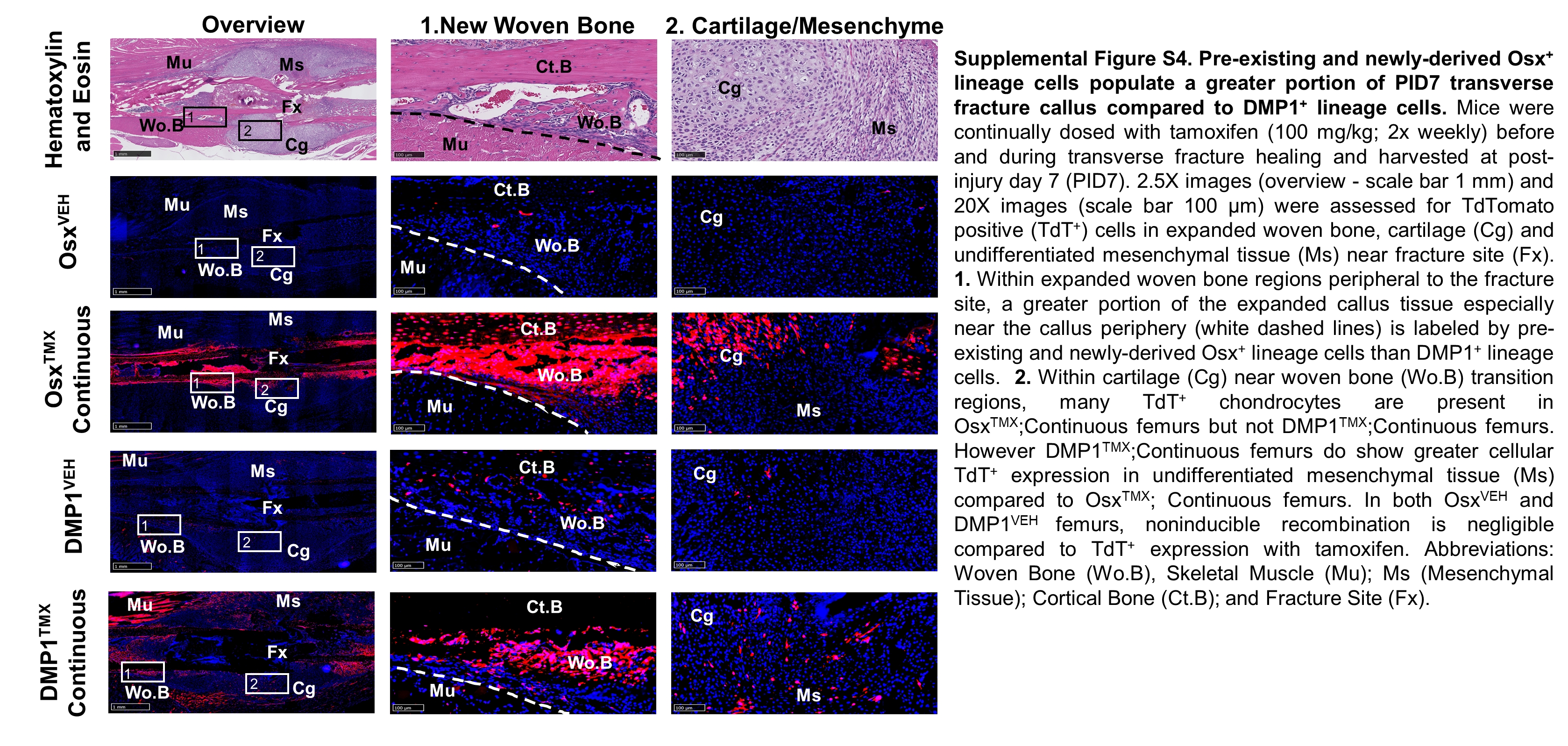

Supplement: Supplementary file 4 [file Image4.jpg]

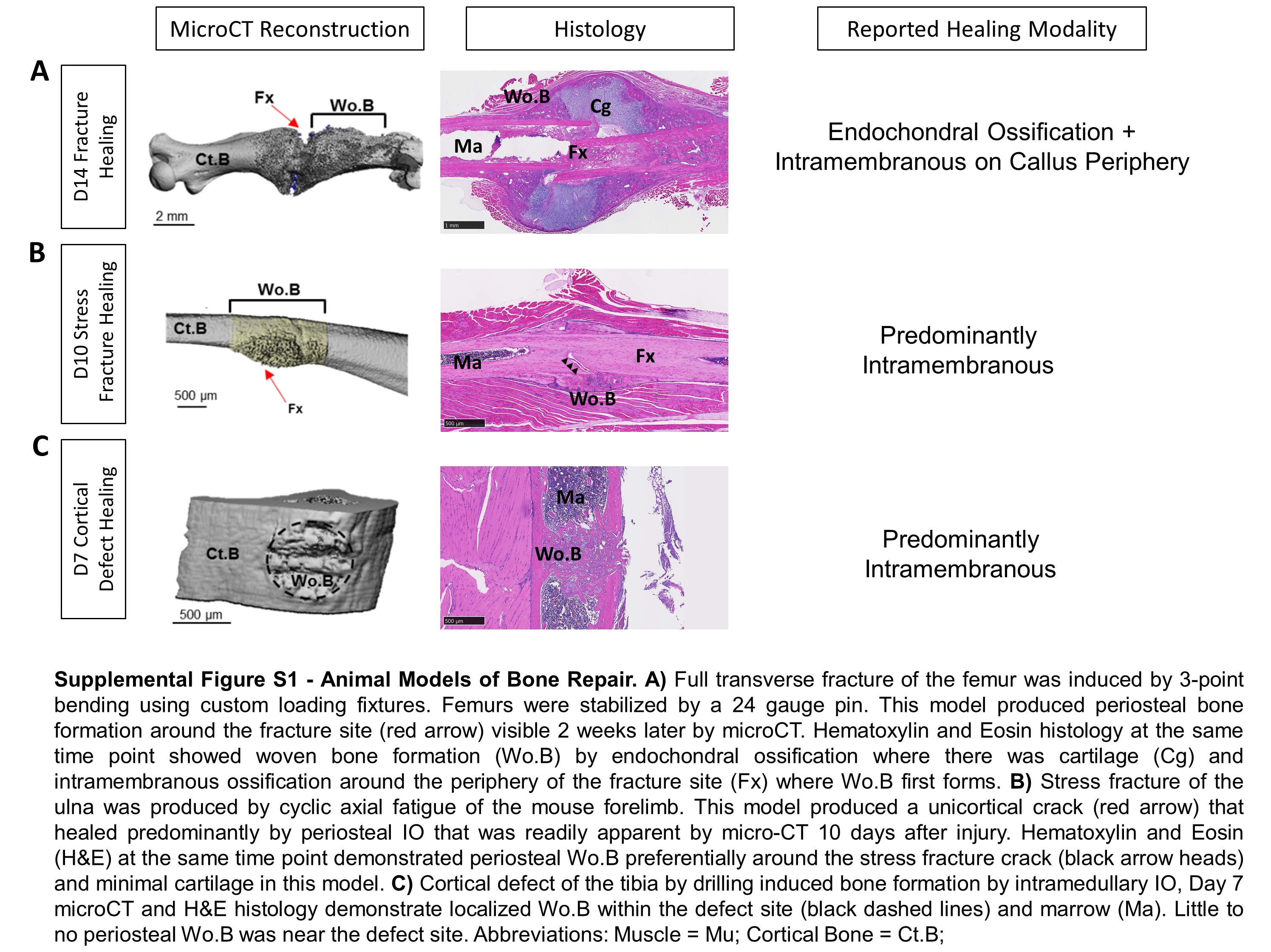

Supplement: Supplementary file 5 [file Image1.jpg]
